# Divergence of Iron Metabolism in Wild Malaysian Yeast

**DOI:** 10.1534/g3.113.008011

**Published:** 2013-10-18

**Authors:** Hana N. Lee, Yulia Mostovoy, Tiffany Y. Hsu, Amanda H. Chang, Rachel B. Brem

**Affiliations:** Department of Molecular and Cell Biology, University of California, Berkeley, California 94720

**Keywords:** iron metabolism, yeast, regulatory variation

## Abstract

Comparative genomic studies have reported widespread variation in levels of gene expression within and between species. Using these data to infer organism-level trait divergence has proven to be a key challenge in the field. We have used a wild Malaysian population of *S. cerevisiae* as a test bed in the search to predict and validate trait differences based on observations of regulatory variation. Malaysian yeast, when cultured in standard medium, activated regulatory programs that protect cells from the toxic effects of high iron. Malaysian yeast also showed a hyperactive regulatory response during culture in the presence of excess iron and had a unique growth defect in conditions of high iron. Molecular validation experiments pinpointed the iron metabolism factors *AFT1*, *CCC1*, and *YAP5* as contributors to these molecular and cellular phenotypes; in genome-scale sequence analyses, a suite of iron toxicity response genes showed evidence for rapid protein evolution in Malaysian yeast. Our findings support a model in which iron metabolism has diverged in Malaysian yeast as a consequence of a change in selective pressure, with Malaysian alleles shifting the dynamic range of iron response to low-iron concentrations and weakening resistance to extreme iron toxicity. By dissecting the iron scarcity specialist behavior of Malaysian yeast, our work highlights the power of expression divergence as a signpost for biologically and evolutionarily relevant variation at the organismal level. Interpreting the phenotypic relevance of gene expression variation is one of the primary challenges of modern genomics.

Heritable genetic differences at the molecular level are widespread between populations and species. Much of the divergence observed in DNA sequence, gene expression, and other molecular attributes may bear little relationship to macroscopic traits, and the outstanding challenge in the field is to detect signatures of biological relevance in catalogs of molecular data. This unbiased search for trait differences with an impact on fitness, motivated by screens of molecular observations alone, is often referred to as reverse ecology (Y. F. Li *et al.* 2008).

The reverse ecology approach is particularly pertinent to the study of regulatory divergence, given that comparisons between species routinely detect expression change in more than half of the genes in a given genome (Wilson and Odom 2009; Dowell 2010). Although linkage or association methods can map the determinants of evolutionarily relevant trait differences within species to loci that affect gene expression (Shapiro *et al.* 2004; Rebeiz *et al.* 2009; Tung *et al.* 2009; Wittkopp *et al.* 2009; Loehlin and Werren 2012), these methods cannot be applied to comparisons between reproductively isolated populations. In landmark cases, focused candidate gene studies have implicated regulatory changes in trait differences between species (Marcellini and Simpson 2006; Hanikenne *et al.* 2008; Booth *et al.* 2010; Werner *et al.* 2010). The ultimate goal is to predict and test phenotypes *ab initio* from genome-scale expression profiles of divergent individuals. To date, little precedent has been set for the experimental validation of such a pipeline.

In this work, we set out to use wild yeast as a model system for the reverse ecology paradigm. Nectar of the flowers of Malaysian bertam palm trees hosts a yeast community whose fermentation is consumed by mammalian pollinators (Wiens *et al.* 2008). *S. cerevisiae* strains collected from this nectar are reproductively isolated from the rest of the species (Cubillos *et al.* 2011), so approaches other than standard mapping methods are required to investigate the evolution of this unique yeast population. An initial unbiased transcriptional profiling experiment led us to the discovery of divergent iron homeostasis and iron-sensitivity behaviors in the Malaysian strains, and to a dissection of the genetic and evolutionary basis of these phenotypes.

## Materials and Methods

### RNA-seq library preparation

All strains used are listed in Supporting Information, Table S2. The three Malaysian (UWOPS03.461.4, UWOPS05.217.3, UWOPS05.227.2) and two wine/European (BC187, RM11-1) isolates used in this study were obtained from the National Collection of Yeast Cultures. For analysis of *cis*-regulatory variation in File S5, the following three Malaysian × wine/European hybrids were generated through single-cell mating between Malaysian and wine/European strains: UWOPS03.461.4 × RM11-1; UWOPS03.461.4 × BC187; and UWOPS05.217.3 × BC187.

Parental and hybrid strains were grown to mid-log phase (0.65–0.72 OD_600_) in yeast peptone dextrose (YPD) medium (Amberg *et al.* 2005) at 30°. Total RNA was extracted from cells using the hot phenol method (Ausubel *et al.* 1995), and genomic DNA was removed with Turbo DNase I (Life Technologies). Libraries were prepared as described by Mortazavi *et al.* (2008) and sequenced on Illumina 2G Genome Analyzer and HiSeq 2000 machines with 100 base-pair paired-end reads.

### RNA-seq

RNA-seq reads from Malaysian and wine/European isolates were mapped against the S288c reference genome (http://www.yeastgenome.org) using Bowtie (Langmead *et al.* 2009). Single nucleotide polymorphisms (SNPs) were called using SAMtools (Li *et al.* 2009) and, for those with the maximum genotype quality Phred score of 99, were used to amend the genome sequences downloaded from Liti *et al.* (2009). These amended genomes were then used as references for re-mapping of RNA-seq reads, with only the reads mapping uniquely and containing no mismatches used for further analysis. For each Malaysian × wine/European hybrid, RNA-seq reads were mapped as described to a genome file formed by concatenation of the amended genomes of the respective parents, yielding only reads that mapped uniquely to regions containing polymorphisms between the Malaysian and wine/European parents and thus reported allele-specific expression.

Read counts per gene were summed using custom Python scripts and normalized with the EDASeq package in R (Risso *et al.* 2011) using the upper-quartile method to correct for differences in library size between lanes and the loess method to correct for differences in GC content within lanes. For analysis of differential expression between wine/European and Malaysian yeast in Table 1 and File S2, for a given gene the multiple isolates from each population were used as replicates in the DESeq package (Anders and Huber 2010) to calculate a composite ratio of the expression levels in the two populations and for assessment of statistical significance, with multiple-testing correction across genes using the Benjamini-Hochberg method. We used the same approach for analysis of allele-specific expression in File S5; for a given gene, read counts per allele from each of the three hybrids were used as input into DESeq to calculate a composite gene expression ratio and significance of the difference between the alleles. As a point of comparison for the calculations of differential expression between parental species and between alleles in the hybrid, we repeated the read-mapping pipeline as described using uncorrected genome sequences from (Liti *et al.* 2009) and re-applied tests for expression change to the estimates from this procedure. At corrected *P* < 0.05, these calculations identified 480 and 0 genes differentially expressed between parents and alleles in the hybrid, respectively (data not shown), in contrast to the 601 and 265 genes, respectively, reported at this significance level in File S2 and File S5.

**Table 1:** Directional expression divergence between Malaysian and wine/European yeast in coregulated gene groups

**Group**	**Annotation**	**Upregulated**	**Adjusted *P***
Node 7	Ribosomal proteins	Malaysian	<0.0001
*AFT1* targets	Iron homeostasis	Wine/European	0.0078
Node 36	Enzymes	Malaysian	0.0156
GO:0007039	Vacuolar protein catabolic process	Malaysian	<0.0001
GO:0006519	Cellular amino acid metabolic process	Wine/European	0.0100
GO:0006725	Cellular aromatic acid metabolic process	Wine/European	0.0133
GO:0006811	Ion transport	Wine/European	0.0300
GO:0006082	Organic acid metabolic process	Wine/European	0.0320
GO:0006826	Iron ion transport	Wine/European	0.0400
GO:0055085	Transmembrane transport	Wine/European	0.0400

Each row reports the results of a test for expression changes of the same sign between Malaysian and wine/European strains in one group of functionally related genes. Group indicates identifier of regulon (Gasch *et al.* 2004) or Gene Ontology term. Upregulated, population with elevated expression. Adjusted *P* indicates significance of a two-sided resampling test relative to the genomic null for an extreme value of the sum, across genes of the indicated regulon, of the signs of the log_2_ ratio of expression in Malaysian yeast to wine/European yeast assessed using all isolates from each population and corrected for multiple testing with the Benjamini-Hochberg method. Shown is the set of tested groups significant at adjusted *P* < 0.05.

### Analysis of directional regulatory divergence in gene groups

To screen regulons for directional expression divergence between Malaysian and wine/European yeast, coregulated gene clusters were obtained from Gasch *et al.* (2004) and Gene Ontology (GO) terms were obtained from the Saccharomyces Genome Database (http://www.yeastgenome.org; April 2010 release). For each regulon, we tabulated all genes whose differential expression between the populations was significant at adjusted *P* < 0.05, and taking the log_2_ fold change between the populations for each such gene as described, we summed these differential expression measures across the genes of the regulon. We then repeated this ratio sum procedure on each of 10,000 random gene groups of the same size as the regulon of interest, drawn from the total set of genes with significant differential expression. The proportion of such null gene groups with a sign sum as extreme as, or more extreme than, the ratio sum of the real regulon was taken to be the nominal empirical *P* for directional expression divergence. *P* values were corrected for multiple testing using the Benjamini-Hochberg method as implemented in the p.adjust() function in R. For analysis of directional *cis*-regulatory variation in File S6, we repeated this group test procedure using as input, for a given gene, the log_2_ ratio of the expression of the Malaysian allele to that of the wine/European allele, measured in hybrids as described above.

**Figure 1:**
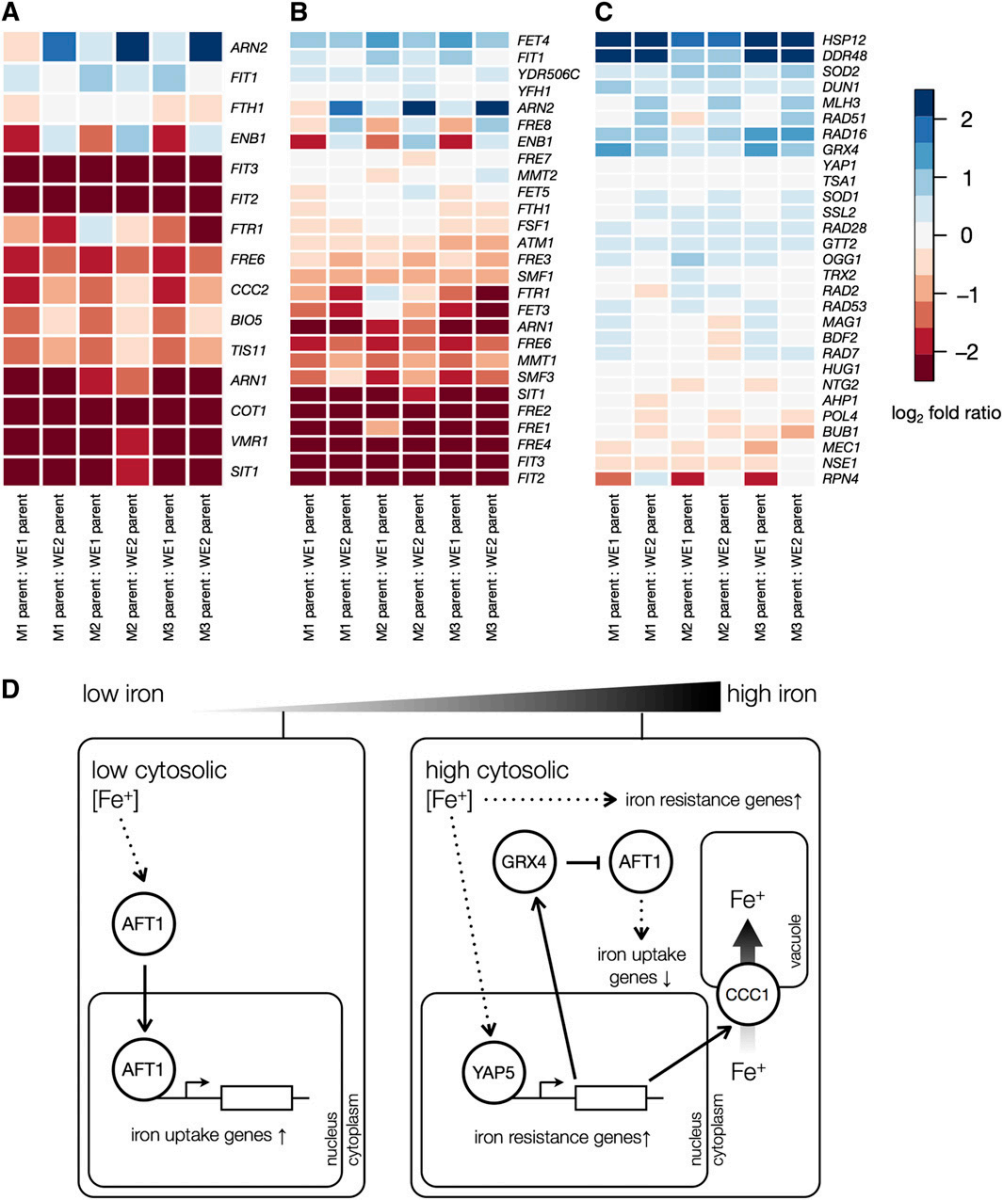
Directional changes in regulon expression between Malaysian and wine/European yeast. (A–C) Each panel shows expression divergence between Malaysian and wine/European yeast in one suite of iron metabolism genes, with the complete set of genes from a previously defined regulon shown in each case. Each cell reports the log_2_ of the expression ratio of one gene between two strains, each column shows data from a comparison between one Malaysian isolate and one wine/European isolate (M1, UWOPS03.461.4; M2, UWOPS05.217.3; M3, UWOPS05.227.2; WE1, RM11-1; WE2, BC187), and each row shows data for one gene. (A) Gene targets of the Aft1p transcription factor (Gasch *et al.* 2004); directional differential expression between populations is significant at corrected *P* = 0.0078 (Table 1). (B) Genes in the Gene Ontology term GO:0006826 iron ion transport (directional expression, *P* = 0.04) (Table 1). (C) Genes upregulated in a *ccc1* laboratory strain compared to wild-type during exposure to high-iron medium (Lin *et al.* 2011) (directional expression, *P* = 0.011) (Table 1). (D) Cartoon of iron homeostasis regulation in laboratory yeast. Under conditions of iron scarcity (left), the transcription factor Aft1p upregulates iron uptake and iron conservation factors. Under conditions of excess iron (right), iron starvation genes are downregulated and the transcription factor Yap5p upregulates iron toxicity response factors, including the vacuolar transporter *CCC1* and other genes functioning in iron storage, iron-sulfur cluster enzyme biogenesis, respiration, DNA damage repair, and oxidative stress response.

To analyze iron-toxicity response genes in Figure 1 and Table 2, we used the set of genes upregulated in a *ccc1* laboratory strain, compared to wild-type, in 3 mM FeSO4 from (Lin *et al.* 2011). Expression analysis and sequence analysis were performed as described. For analysis of iron toxicity response factors in Figure 4 and Figure S2, we also mined the set of genes responsive to 2 mM FeSO_4_ in (Pimentel *et al.* 2012).

**Table 2:** Elevated protein evolutionary rates in iron toxicity response genes in Malaysian yeast

**Population**	**Regulon Mean K_a_/K_s_**	**Genome Mean K_a_/K_s_**	***P***
Wine/European	0.1469	0.1240	0.2871
Malaysian	0.2629	0.1650	0.0416
Sake	0.0594	0.1014	0.7481
North American	0.0487	0.0822	0.7511
West African	0.1878	0.1777	0.3861

Each row reports analysis of the protein evolutionary rate, in one yeast population, of the set of genes upregulated in a *ccc1* laboratory strain compared to wild-type during exposure to high-iron medium (Lin *et al.* 2011). *P*, the significance of a one-sided resampling test relative to the genomic null for a greater value of the mean, across genes of the iron toxicity response regulon, of ratios of nonsynonymous (K_a_) to synonymous (K_s_) coding sequence variants private to and fixed in the indicated yeast population.

### Reciprocal hemizygote strain construction

Malaysian × wine/European hybrid strains (YHL243 and YHL247, both UWOPS03.461.4 × BC187 hybrids from reciprocal matings) (Table S2) were used to make reciprocal hemizygotes by deleting one allele of the gene of interest (*CCC1*, *YAP5*, or *AFT1*) with a *URA3* cassette (Sikorski and Hieter 1989). The targeted locus was sequenced in each transformant to verify that only one allele was present and to identify the allele.

### Growth assays

**Figure 2:**
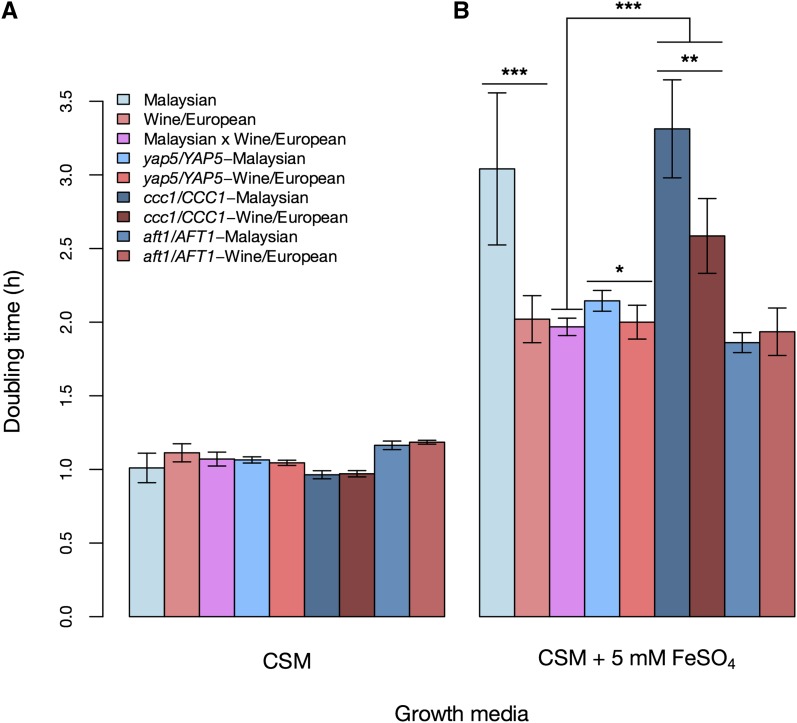
*YAP5* and *CCC1* underlie growth defects of Malaysian yeast in high-iron conditions. Each bar reports the mean doubling time of one yeast strain during log-phase growth in the indicated condition. In each panel, Malaysian and wine/European indicate the average growth across biological replicates of the Malaysian strain UWOPS03.461.4 (n = 9) and the wine/European strain BC187 (n = 9), respectively. Malaysian × wine/European indicates growth of the UWOPS03.461.4 × BC187 hybrid (n = 15). The fourth and fifth bars of each panel report growth of *YAP5* reciprocal hemizygotes in the UWOPS03.461.4 × BC187 hybrid background, with *yap5/YAP5*-Malaysian denoting strains harboring only the allele of *YAP5* from the Malaysian strain (n = 12), and *yap5/YAP5*-wine/European denoting the strains with the wine/European allele of *YAP5* (n = 6). The sixth and seventh bars report growth of *CCC1* reciprocal hemizygotes in the hybrid background, with *ccc1/CCC1*-Malaysian denoting hemizygotes harboring only the allele of CCC1 from the Malaysian strain (n = 12), and *ccc1/CCC1*-wine/European denoting hemizygotes with the wine/European allele of *CCC1* (n = 6). The last two bars report growth of *AFT1* reciprocal hemizygotes in the hybrid background, with *aft1/AFT1*-Malaysian denoting hemizygotes harboring only the allele of *AFT1* from the Malaysian strain (*n* = 6), and *aft1/AFT1*-wine/European denoting hemizygotes with the wine/European allele of *AFT1* (n = 6). Error bars in all panels represent 95% CIs. (A) Complete media without additional FeSO_4_. (B) Complete media supplemented with 5 mM FeSO_4_. ***Significant at Wilcoxon *P* < 0.0005; **Wilcoxon *P* < 0.005; *Wilcoxon *P* < 0.05. Raw measurements are reported in File S3.

For each strain, a preculture was prepared by growth for 4 hr in YPD medium and diluted to 0.1 OD_600_ by resuspension in distilled water. Experimental cultures were then established in triplicate for each strain by diluting this preculture 1:10 in complete synthetic medium (Amberg *et al.* 2005) supplemented with 5 mM FeSO_4_ or supplemented with an equal amount of water as an untreated control for a starting concentration of 0.01 OD_600_ in a 96-well plate to assess growth. Growth was monitored via OD_600_ measurements every 30 min in a Tecan GENios microplate reader. Growth attributes in Figure 2, File S3, and File S4 were calculated from growth curves using the method of Warringer *et al.* (2011) implemented in Python. In Figure 2 and File S3, culturing and analysis were performed for two to four transformants of each reciprocal hemizygote.

### Quantitative PCR

Two biological replicates of each parental strain, two biological replicates of each of two independent transformants of each *AFT1* and *YAP5* reciprocal hemizygote, and two sets of *CCC1* reciprocal hemizygote strains, each comprising two biological replicates of each of two independent transformants, were grown to mid-log phase in YPD medium at 30°. For iron-treated samples, FeSO_4_ was added to a final concentration of 5 mM. After 30 min of incubation at 30°, total RNA was isolated and treated with DNase I as described for RNA-seq, except that for iron-treated samples, RNA was purified on RNeasy columns (Qiagen). For each sample, cDNA was synthesized using SuperScript III reverse transcriptase (Life Technologies) and diluted to 1 ng/µL. Maxima SYBR green (Thermo Scientific) was used for quantitative PCR, with two to three technical replicates for each gene in each strain, on the Mx3000P system (Agilent). Cycle thresholds (Ct) for each replicate were obtained using the MxPro software (Agilent), and gene expression levels relative to *ACT1* were calculated using the 2^−ΔΔCt^ method (Livak and Schmittgen 2001). As a further normalization step for ease of comparison between experiments, the vector of expression measures of a given gene in a given strain background and replicate set was centered with respect to the median across the set. Statistical significance of differential expression between genotypes for a given regulon was assessed by a paired Wilcoxon test. *YAP5* reciprocal hemizygotes did not differ significantly with respect to iron starvation gene expression, and we detected little impact of variation at *AFT1*, *YAP5*, or *CCC1* on iron resistance genes in synthetic complete medium (data not shown).

### K_a_/K_s_ analysis

Multiple sequence alignments were generated for each gene using MUSCLE (Edgar 2004) from genome sequences of all nonmosaic strains as defined by Liti *et al.* (2009) using uncorrected published sequences. The ratio of nonsynonymous to synonymous substitution rates, using the formula for K_a_/K_s_ described by Nei and Gojobori (1986), was calculated for each gene using SNPs fixed in and private to each nonmosaic yeast population, with populations as defined by Liti *et al.* (2009). For each population, we calculated the mean of these K_a_/K_s_ values across all genes of the iron toxicity response regulon as defined. We then repeated this mean K_a_/K_s_ calculation on each of 10,000 random gene groups of the same size as the iron toxicity response regulon, drawn from all genes for which SNP data were available. The proportion of such null gene groups with a mean as extreme as, or more extreme than, the mean of the real regulon was taken to be the empirical *P* value for protein evolutionary rate.

## Results

To survey gene expression programs in wild yeast, we took a comparative approach using three homozygous Malaysian isolates and two homozygotes from a distinct, well-defined wine/European yeast population (Liti *et al.* 2009). We cultured each strain in rich medium and subjected each to transcriptional profiling by RNA-seq (Table S1). We used the RNA-seq reads to verify and correct coding sequences from low-coverage genomes available for these strains (Liti *et al.* 2009), identifying a total of 46,367 SNPs in coding regions in which all Malaysian strains sequenced harbored one allele and all wine/European strains harbored a second allele (File S1). Mapping to the amended genomes and analysis of the complete set of profiles revealed 601 genes differentially expressed between the populations at a false discovery rate (FDR) of 5% (File S2).

We sought to focus on patterns of regulatory variation across groups of functionally related genes, which could be a signpost for organismal trait differences between Malaysian and wine/European yeast. For this purpose, we screened gene groups for coherent expression change between the populations. Testing groups delineated by GO (http://yeastgenome.org) and those defined on the basis of coregulation in classic analyses of laboratory yeast (Gasch *et al.* 2004), we identified seven GO terms and three regulons whose genes were predominantly upregulated, or predominantly downregulated, in Malaysian strains relative to wine/European strains (FDR = 5%) (Table 1). Among these were two sets of genes annotated in iron metabolism for which the Malaysian population was associated with low expression (Figure 1, A and B): targets of Aft1p, a transcription factor regulating iron uptake genes, and the iron ion transport GO term GO:0006826. This largely nonoverlapping pair of gene groups comprises genes that, in studies of laboratory yeast, are expressed at low levels in the presence of excess iron, at moderate levels in standard medium, and at high levels during iron starvation (Yamaguchi-Iwai *et al.* 1996, 2002; Shakoury-Elizeh *et al.* 2004) (Figure 1D).

Given the divergent expression of iron starvation genes between Malaysian and wine/European yeasts, we hypothesized that regulation of factors that protect against the toxic effects of excess iron might also be distinct between these two populations. To test this, we used a previously defined suite of genes upregulated by laboratory yeast in iron-toxic conditions (Lin *et al.* 2011), which includes components of the DNA damage and oxidative stress response pathways that we refer to as iron resistance genes. Although, as expected, in the absence of excess iron this regulon was not highly expressed in either population, mRNA levels were modestly but significantly elevated in the Malaysian population (Figure 1C). Thus, Malaysian yeast cultured in standard laboratory conditions repressed iron uptake genes and activated genes involved in resistance to iron toxicity relative to wine/European isolates (Figure 1D).

**Figure 3:**
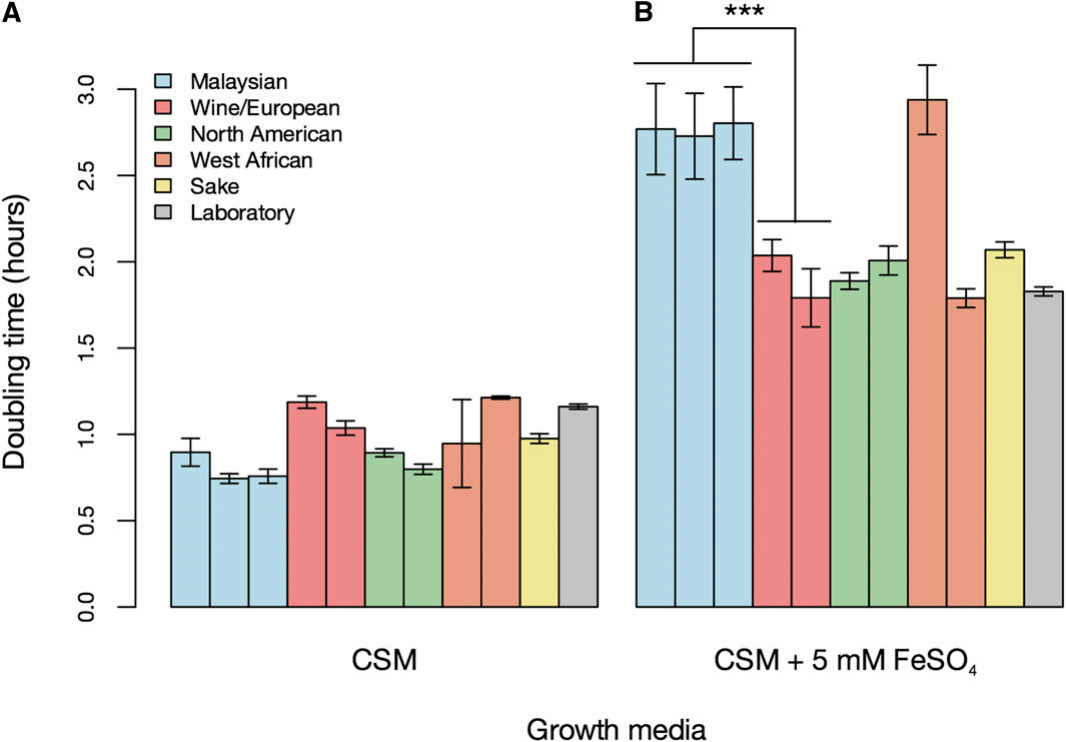
Most non-Malaysian yeast isolates are resistant to high-iron toxicity. Each bar reports the mean doubling time of one yeast strain (n = 3) during log-phase growth in the indicated condition. Each color reports growth of strains from one yeast population as defined by Liti *et al.* (2009): blue, Malaysian (from left to right, strains UWOPS03.461.4, UWOPS05.217.3, UWOPS05.227.2); red, wine/European (BC187, RM11-1); green, North American (YPS606, YPS128); orange, West African (NCYC110, DBVPG6044); yellow, sake (Y12); gray, laboratory (S288C). (A) Complete media without FeSO_4_. (B) Complete media with 5 mM FeSO_4_. ***Significant at Wilcoxon *P* < 0.0005. Error bars represent 95% CIs. Raw measurements are reported in File S4.

We hypothesized that the molecular differences in iron metabolism between yeast populations could have organism-level correlates in the context of growth during iron challenge. To test this, we first measured growth rates of Malaysian and wine/European parent strains, as well as the wild-type hybrid formed by a mating between them, in iron toxicity conditions and in a complete medium control. In the presence of excess iron, Malaysian parent strains had a marked growth defect, with doubling times increased up to 1.7-fold relative to wine/European isolates (Figure 2 and File S3). This defect acted as a recessive trait, because hybrid strains showed resistance to high-iron treatment on par with that of their wine/European parents (Figure 2 and File S3). We also assessed growth of other environmental yeast isolates and found all to be resistant to iron toxicity (Figure 3 and File S4), with the exception of NCYC110, a member of the broadly stress-sensitive West African population (Zorgo *et al.* 2012).

We next sought to dissect the genetic basis of divergence in iron metabolism regulation among Malaysian strains using a candidate gene framework. We expected that causative variants would likely act in *trans* on the iron metabolism machinery, because measurements of allele-specific expression in Malaysian × wine/European hybrids showed no strong signals of coherent *cis*-regulatory variation in iron metabolism genes (Table S1, File S5, and File S6). Likewise, sequence-based analyses revealed no striking promoter variation in iron metabolism genes (data not shown). To identify candidate *trans*-acting loci underlying growth and regulatory traits, we searched the genome for nonsynonymous coding polymorphisms unique to the Malaysian population in components of the iron metabolism gene network. Among the resulting gene hits, we focused on two known to be essential in laboratory yeast for growth in high-iron conditions (Li *et al.* 2001; L. Li *et al.* 2008): the transcription factor *YAP5*, which activates genes in response to treatment with excess iron, and *CCC1*, a transporter that sequesters iron ions in the vacuole (Figure 1D). We also chose for molecular validation the transcription factor *AFT1*, a protein that has been characterized for its activity in low levels of iron as a regulator of the iron-scavenging genes emerging from our original RNA-seq analysis (Shakoury-Elizeh *et al.* 2004) (Figure 1, A and D). In all three protein sequences, we observed nonconservative amino acid changes in Malaysian strain genomes (Figure S1).

To investigate the role of our candidate genes in the Malaysian growth phenotype in high iron, we used reciprocal hemizygote analysis (Steinmetz *et al.* 2002) for each candidate locus as follows. In the diploid hybrid formed by mating a haploid Malaysian strain to a haploid wine/European, we knocked out each allele in turn of the gene of interest. The resulting hybrid strains in such a pair were isogenic to one another throughout the genome except at the hemizygous locus, where each strain harbored only one of the two alleles from the parent strains. Any phenotypic differences between the strains of a hemizygote pair could thus be ascribed to variation at the manipulated site. We cultured reciprocal hemizygote strains for *AFT1*, *YAP5*, and *CCC1* in high-iron conditions and in a complete medium control, along with the wild-type parents, and we measured growth rates of each strain in each case. The results revealed a 1.2-fold difference in iron resistance between strains bearing the Malaysian and wine/European alleles of *CCC1* and a more modest (1.05-fold), although still significant, effect on iron resistance of variation in *YAP5* (Figure 2 and File S3). In each case, the Malaysian allele of the respective gene compromised growth in high-iron conditions, mirroring the known effects of engineered null mutations at these loci (Li *et al.* 2001; L. Li *et al.* 2008).

**Figure 4:**
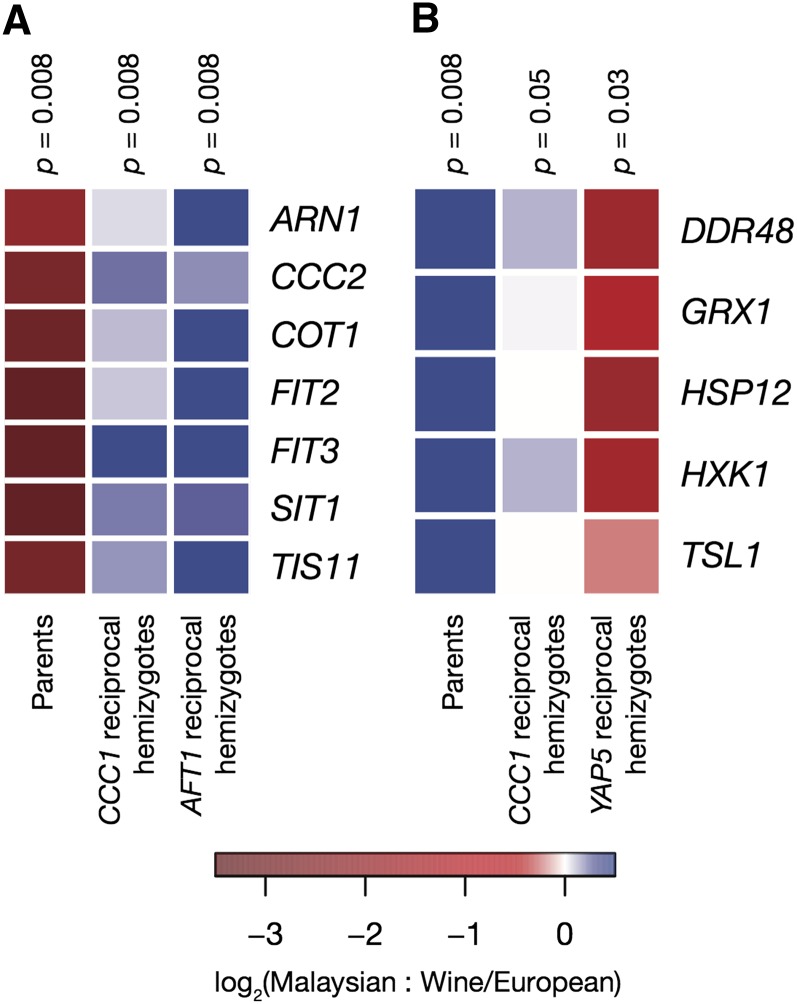
Regulatory impact of variation in *AFT1*, *CCC1*, and *YAP5* between Malaysian and wine/European yeast. Each panel reports the regulatory effects of variation at iron metabolism genes in yeast cultured in one growth medium. In a given panel, each row reports comparisons between the effects of Malaysian and wine/European genotypes on expression of an iron starvation (Gasch *et al.* 2004) or iron resistance (Lin *et al.* 2011; Pimentel *et al.* 2012) target gene. Color in each cell represents a ratio of the expression measurements from two strains. A given cell in the first column reports the ratio, for the indicated target gene, of the expression in a homozygous Malaysian strain (UWOPS03.461.4), as a median across replicates (n = 2), to that in a homozygous wine/European strain (BC187). A given cell in each remaining column reports expression as a median across replicates in a Malaysian-wine/European hemizygote (UWOPS03.461.4 × BC187) bearing the Malaysian allele of the indicated variant locus (*CCC1*, n = 8; *AFT1*, n = 4; *YAP5*, n = 4) relative to the median expression in the hemizygote bearing the wine/European allele. (A) Iron starvation genes in synthetic complete medium. (B) Iron resistance genes in synthetic complete medium supplemented with 5 mM FeSO_4_. Values at the top of each column report the results of a paired Wilcoxon test for the significance of the differences in expression, across genes of the regulon, between the indicated genotypes. Individual replicate measurements are reported in Figure S2 and raw data are reported in File S7 and File S8.

Expression measurements also supported a model of losses of function at Malaysian orthologs of *CCC1* and *YAP5*: the Malaysian allele of *CCC1* drove upregulation of iron starvation genes and iron resistance genes in standard complete medium and high-iron conditions, respectively, whereas the Malaysian allele of *YAP5* reduced expression of iron resistance genes in high-iron conditions (Figure 4, Figure S2, File S7, and File S8), consistent with the behavior of hypomorphs at these loci (Li *et al.* 2001; L. Li *et al.* 2008; Lin *et al.* 2011). Additionally, strains hemizygous for *CCC1* grew more slowly than wild-type hybrids in high-iron medium regardless of which allele they carried (Figure 2 and File S3), reflecting haploinsufficiency of *CCC1* with respect to iron resistance. As expected, variation in *AFT1*, which functions primarily in conditions of iron starvation (Figure 1D), had little impact on growth when iron was present in excess (Figure 2 and File S3). However, the Malaysian allele of *AFT1* had a significant regulatory effect in reciprocal hemizygotes, driving upregulation of iron starvation genes during growth in complete medium (Figure 4, Figure S2, and File S7) in a manner consistent with a gain-of-function mutation (Shakoury-Elizeh *et al.* 2004). We conclude that the growth defect of Malaysian yeast in the presence of excess iron is attributable, in part, to alleles that act at losses of function at the iron resistance genes *YAP5* and *CCC1*. With respect to gene regulation, the Malaysian genome harbors alleles with both activating and repressing effects on iron metabolism, reflecting a complex genetic model that involves *YAP5* and *CCC1* as well as *AFT1*.

We next aimed to investigate signatures of natural selection on iron metabolism in Malaysian yeast, complementing our expression-based and phenotype-based analyses with sequence-based tests. Using SNPs private to and conserved in each of five yeast populations characterized by Liti *et al.* (2009), we estimated ratios of nonsynonymous to synonymous substitution rates in coding regions of iron metabolism genes and compared these rates to those of the rest of the genome. The results, shown in Table 2, revealed a 1.6-fold elevation of protein evolutionary rate in Malaysian yeast in genes activated during high-iron exposure, with no such effect detectable in any other yeast population. Iron starvation genes exhibited no signature of non-neutral sequence divergence in any population (data not shown). These results provide evidence for a unique history of a change of selective pressure on iron resistance genes in Malaysian yeast, lending further support to the notion of derived iron-response behaviors in the Malaysian population.

## Discussion

Against a backdrop of hundreds of comparative transcriptomic studies in many species, the power of expression divergence as a predictor of trait variation has remained largely invalidated. To establish Malaysian yeast as a test bed for the reverse ecology approach, we generated expression profiles from cultures grown in standard conditions, and we used these data as a point of departure for a study of regulatory and growth phenotypes in the presence of excess iron. Although several elegant studies have characterized growth traits in yeast populations (Warringer *et al.* 2011; Hodgins-Davis *et al.* 2012; Zorgo *et al.* 2012), to date the degree of divergence between strains in iron sensitivity has been unknown. Our expression measurements, growth assays, and molecular genetic manipulations confirmed the iron sensitivity of Malaysian yeast and the role of three candidate genes, *AFT1*, *YAP5*, and *CCC1*, in regulatory and growth phenotypes.

What are the evolutionary forces that have driven divergence of iron metabolism in Malaysian yeast? The behavior of the Malaysian *YAP5* and *CCC1* alleles as losses of function, and the sequence-based evidence for non-neutral evolution of the coding regions of iron resistance genes, support a model of specialization by Malaysian strains to a low-iron environment. In one evolutionary scenario, reduced exposure to high-iron conditions in the Malaysian niche would have relaxed the strength of purifying selection on the iron toxicity response, allowing Malaysian yeasts to accumulate mutations in this gene network. Additionally, given the gain-of-function behavior of the Malaysian allele of *AFT1*, it is tempting to speculate that negative regulatory control of this activator of the iron starvation response has been eliminated in Malaysian yeast to sidestep iron-sensing mechanisms and raise expression of iron transporters and scavengers as would be suitable for a constant, low-iron environment. By contrast, the avid iron uptake and functional vacuolar iron storage of wine/European strains would reflect a need for flexible iron homeostasis machinery that can respond more fully to environmental change. Taken together, our findings provide a case in which the rare and often deleterious mutations that litter wild yeast genomes (Zorgo *et al.* 2012) follow a compelling evolutionary logic. A broader involvement of additional metals is suggested by the growth defect of a Malaysian isolate in high-copper medium (Hodgins-Davis *et al.* 2012). The emerging picture is one in which the Malaysian yeast population has experienced unique evolutionary pressures on metal metabolism, highlighting the palm flower niche of these microbes as a driver of evolutionary change.

Although we successfully harnessed expression profiles to identify an evolutionarily relevant trait difference between yeast populations, our results leave open the question of the molecular basis of the expression patterns themselves. In reciprocal hemizygote experiments, the most striking regulatory effects of our candidate genes were in directions that opposed the patterns of divergence in parental homozygotes: Malaysian alleles at the *YAP5* and *AFT1* transcription factors drove downregulation of iron resistance genes and upregulation of iron starvation genes, respectively, contrasting with the high and low expressions of these gene sets, respectively, in Malaysian parental strains. Given the prevalence of transgressive segregation in the genetics of gene expression variation within species (Gibson and Weir 2005), we favor a model in which the regulatory program of Malaysian homozygotes is an indirect regulatory response to a suite of defects in iron resistance genes in Malaysian homozygotes, one which obscures the transgressive contribution of *YAP5* and *AFT1* in the purebred context. However, our experiments do not rule out the possibility that alleles of *YAP5* and *AFT1* exert different effects in the parental background than they do in the hybrid. Future work will establish the potential for epistasis among our candidate loci and between each gene and the genetic background using the loci we have validated here as a springboard to deepen our mechanistic understanding of the regulatory and growth behaviors of Malaysian isolates.

Our work makes clear that expression profiles can be used as a powerful hypothesis generator for the study of organismal trait variation. This reverse ecology paradigm is likely to be most successful when coherent regulatory change in well-annotated pathways underlies, or responds to, change in a phenotype. With the increased availability of functional genomic resources in many taxa, the expression-based approach will likely prove to be broadly applicable in the genetic dissection of differences between populations and species.

## Supplementary Material

Supporting Information
