# Supplementary material for: Divergence of Iron Metabolism in Wild Malaysian Yeast
Source: G3 (Bethesda). 2013 Oct 18;3(12):2187–94. doi: 10.1534/g3.113.008011 (PMC3852381; doi:10.1534/g3.113.008011)
Supplement: Supporting Information [file supp_3_12_2187__index.html]

Divergence of Iron Metabolism in Wild Malaysian Yeast — Supporting Information 

# Divergence of Iron Metabolism in Wild Malaysian Yeast

## Supporting Information for Lee *et al.*, 2013

**Files in this Data Supplement:**

- Supporting Information - Figures S1-S2, Files S1-S8, and Tables S1-S2 (PDF, 1 MB)
- Figure S1 - Coding variants private to Malaysian yeast strains in iron-metabolism genes. (PDF, 1 MB)
- Figure S2 - Regulatory impact of variation in *AFT1, CCC1,* and *YAP5* between Malaysian and wine/European yeast. (PDF, 444 KB)
- Table S1 - RNA-seq statistics. (PDF, 313 KB)
- Table S2 - Strains used in this work. (PDF, 325 KB)
- Contents of Files S1-S8 - Complete descriptions of the contents of Files S1-S8 (PDF, 434 KB)
- File S1 - Single-nucleotide polymorphisms in Malaysian and wine/European coding sequences inferred from RNA-seq (.zip, 1017 KB)
- File S2 - Expression profiles of Malaysian and wine/European yeast, measured by RNA-seq (.zip, 339 KB)
- File S3 - Expression profiles of hybrid strains formed by a mating between Malaysian and wine/European yeast, measured by RNA-seq (.zip, 4 KB)
- File S4 - Directional *cis*-regulatory variation between Malaysian and wine/European yeast in co-regulated gene groups (.zip, 2 KB)
- File S5 - Expression of iron-starvation genes in Malaysian and wine/European parent strains and reciprocal hemizygotes in standard conditions, measured by quantitative PCR (.zip, 316 KB)
- File S6 - Expression of iron-resistance genes in Malaysian and wine/European parent strains and reciprocal hemizygotes in high-iron conditions, measured by quantitative PCR (.zip, 26 KB)
- File S7 - Growth attributes, in standard conditions and in high iron, of Malaysian and wine/European yeast, their hybrids, and reciprocal hemizygotes for *AFT1, YAP5,* and *CCC1* (.zip, 3 KB)
- File S8 - Growth attributes, in standard conditions and in high iron, of a panel of environmental yeast isolates (.zip, 2 KB)
