# Supplementary material for: Divergence of Iron Metabolism in Wild Malaysian Yeast
Source: G3 (Bethesda). 2013 Oct 18;3(12):2187–94. doi: 10.1534/g3.113.008011 (PMC3852381; doi:10.1534/g3.113.008011)
Supplement: Supporting Information [file supp_g3.113.008011_008011SI.pdf]

G3·Genes | Genomes | Genetics

Divergence of iron metabolism in wild Malaysian yeast

Hana N. Lee[¶^], Yulia Mostovoy[^], Tiffany Y. Hsu[§], Amanda H. Chang, and Rachel B. Brem*

Department of Molecular and Cell Biology, University of California, Berkeley
[¶]Current address: Department of Ecology and Evolution, University of Chicago, Chicago, IL
[§]Current address: Graduate Program in Biological and Biomedical Sciences, Harvard Medical School, Boston, MA
[^]These authors contributed equally to this work.
*To whom correspondence should be addressed: rbrem@berkeley.edu

DOI: 10.1534/g3.113.008011

A

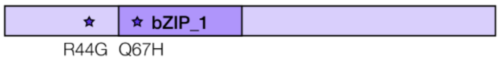


```
YPS606           21   KIHVSKNWKLPPRLPHRAAQRRKRVHRLHEDYETEENDEELQKKKRQNRDAQRAYRERKN   80
Y12              21   KIHVSKNWKLPPRLPHRAAQRRKRVHRLHEDYETEENDEELQKKKRQNRDAQRAYRERKN   80
YPS128           21   KIHVSKNWKLPPRLPHRAAQRRKRVHRLHEDYETEENDEELQKKKRQNRDAQRAYRERKN   80
Y9               21   KIHVSKNWKLPPRLPHRAAQRRKRVHRLHEDYETEENDEELQKKKRQNRDAQRAYRERKN   80
DBVPG6765        21   KIHVSKNWKLPPRLPHRAAQRRKRVHRLHEDYETEENDEELQKKKRQNRDAQRAYRERKN   80
BC187            21   KIHVSKNWKLPPRLPHRAAQRRKRVHRLHEDYETEENDEELQKKKRQNRDAQRAYRERKN   80
YJM981           21   KIHVSKNWKLPPRLPHRAAQRRKRVHRLHEDYETEENDEELQKKKRQNRDAQRAYRERKN   80
DBVPG1106        21   KIHVSKNWKLPPRLPHRAAQRRKRVHRLHEDYETEENDEELQKKKRQNRDAQRAYRERKN   80
DBVPG1373        21   KIHVSKNWKLPPRLPHRAAQRRKRVHRLHEDYETEENDEELQKKKRQNRDAQRAYRERKN   80
YJM978           21   KIHVSKNWKLPPRLPHRAAQRRKRVHRLHEDYETEENDEELQKKKRQNRDAQRAYRERKN   80
S288c            21   KIHVSKNWKLPPRLPHRAAQRRKRVHRLHEDYETEENDEELQKKKRQNRDAQRAYRERKN   80
YJM975           21   KIHVSKNWKLPPRLPHRAAQRRKRVHRLHEDYETEENDEELQKKKRQNRDAQRAYRERKN   80
L_1528           21   KIHVSKNWKLPPRLPHRAAQRRKRVHRLHEDYETEENDEELQKKKRQNRDAQRAYRERKN   80
RM11_1A          21   KIHVSKNWKLPPRLPHRAAQRRKRVHRLHEDYETEENDEELQKKKRQNRDAQRAYRERKN   80
DBVPG1788        21   KIHVSKNWKLPPRLPHRAAQRRKRVHRLHEDYETEENDEELQKKKRQNRDAQRAYRERKN   80
L_1374           21   KIHVSKNWKLPPRLPRRAAQRRKRVHRLHEDYETEENDEELQKKKRQNRDAQRAYRERKN   80
UWOPS05_227_2    21   KIHVSKNWKLPPRLPHRAAQRRKGVHRLHEDYETEENDEELQKKKRHNRDAQRAYRERKN   80
UWOPS05_217_3    21   KIHVSKNWKLPPRLPHRAAQRRKGVHRLHEDYETEENDEELQKKKRHNRDAQRAYRERKN   80
UWOPS03_461_4    21   KIHVSKNWKLPPRLPHRAAQRRKGVHRLHEDYETEENDEELQKKKRHNRDAQRAYRERKN   80
DBVPG6044        21   KIHVSKNWKLPPRLPHRAAQRRKRVHRLHEDYETEENDEELQKKKRQNRDAQRAYRERQN   80
NCYC110          21   KIHVSKNWKLPPRLPHRAAQRRKRVHRLHEDYETEENDEELQKKKRQNRDAQRAYRERQN   80
S. paradoxus     21   KIHVSKNWKLPPRLPHRATQRRKRAHRLHEEYETEGNDEALQKKKRQNRDAQRAYRERKN   80
```

B

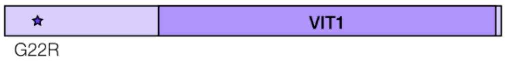


```
UWOPS05_227_2     1   MSIVALKNAVVTLIQKAKGSGRTSELGGSESTPLLRGSNSNSSRHDNLSSSSSDIIYGRN   60
UWOPS05_217_3     1   MSIVALKNAVVTLIQKAKGSGRTSELGGSESTPLLRGSNSNSSRHDNLSSSSSDIIYGRN   60
UWOPS03_461_4     1   MSIVALKNAVVTLIQKAKGSGRTSELGGSESTPLLRGSNSNSSRHDNLSSSSSDIIYGRN   60
DBVPG6765         1   MSIVALKNAVVTLIQKAKGSGGTSELGGSESTPLLRGSNSNSSRHDNLSSSSSDIIYGRN   60
DBVPG1788         1   MSIVALKNAVVTLIQKAKGSGGTSELGGSESTPLLRGSNSNSSRHDNLSSSSSDIIYGRN   60
RM11_1A           1   MSIVALKNAVVTLIQKAKGSGGTSELGGSESTPLLRGSNSNSSRHDNLSSSSSDIIYGRN   60
L_1528            1   MSIVALKNAVVTLIQKAKGSGGTSELGGSESTPLLRGSNSNSSRHDNLSSSSSDIIYGRN   60
YJM789            1   MSIVALKNAVVTLIQKAKGSGGTSELGGSESTPLLRGSNSNSSRHDNLSSSSSDIIYGRN   60
YJM975            1   MSIVALKNAVVTLIQKAKGSGGTSELGGSESTPLLRGSNSNSSRHDNLSSSSSDIIYGRN   60
L_1374            1   MSIVALKNAVVTLIQKAKGSGGTSELGGSESTPLLRGSNSNSSRHDNLSSSSSDIIYGRN   60
S288c             1   MSIVALKNAVVTLIQKAKGSGGTSELGGSESTPLLRGSNSNSSRHDNLSSSSSDIIYGRN   60
YPS606            1   MSIVALKNAVVTLIQKAKGSGGTSELGGSESTPLLRGSNSNSSRHDNLSSSSSDIIYGRN   60
Y12               1   MSIVALKNAVVTLIQKAKGSGGTSELGGSESTPLLRGSNSNSSRHDNLSSSSSDIIYGRN   60
YPS128            1   MSIVALKNAVVTLIQKAKGSGGTSELGGSESTPLLRGSNSNSSRHDNLSSSSSDIIYGRN   60
YJM978            1   MSIVALKNAVVTLIQKAKGSGGTSELGGSESTPLLRGSNSNSSRHDNLSSSSSDIIYGRN   60
Y9                1   MSIVALKNAVVTLIQKAKGSGGTSELGGSESTPLLRGSNSNSSRHDNLSSSSSDIIYGRN   60
NCYC110           1   MSIVALKNAVVTLIQKAKGSGGTSELGGSESTPLLRGSNSNSSRHDNLSSSSSDIIYGRN   60
DBVPG1373         1   MSIVALKNAVVTLIQKAKGSGGTSELGGSESTPLLRGSNSNSSRHDNLSSSSSDIIYGRN   60
YJM981            1   MSIVALKNAVVTLIQKAKGSGGTSELGGSESTPLLRGSNSNSSRHDNLSSSSSDIIYGRN   60
BC187             1   MSIVALKNAVVTLIQKAKGSGGTSELGGSESTPLLRGSNSNSSRHDNLSSSSSDIIYGRN   60
DBVPG1106         1   MSIVALKNAVVTLIQKAKGSGGTSELGGSESTPLLRGSNSNSSRHDNLSSSSSDIIYGRN   60
DBVPG6044         1   MSIVALKNAVVTLIQKAKGSGGTSELGGSESTPLLRGSNSNSSRHDNLSSSSSDIIYGRN   60
S. paradoxus      1   MSIVALKNAVVTLIQKAKGSGGTSDLGGSESTPLLRGSNSNSSRHDNLSSSSNSDIIYGRN  60
```

C

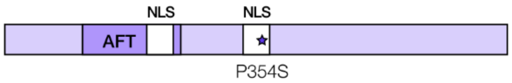


```
K11             301   SFNVVLPTNSNVTSSASSSTVSSISLDSSNASKRPCLPSVNNTGSINTNNVRKPKSQCKN  360
Y12             301   SFNVVLPTNSNVTSSASSSTVSSISLDSSNASKRPCLPSVNNTGSINTNNVRKPKSQCKN  360
Y9              301   SFNVVLPTNSNVTSSASSSTVSSISLDSSNASKRPCLPSVNNTGSINTNNVRKPKSQCKN  360
NCYC110         301   SFNVVLPTNSNVTSSASSSTVSSISLDSSNASKRPCLPSVNNTGSINTNNVRKPKSQCKN  360
DBVPG6044       301   SFNVVLPTNSNVTSSASSSTVSSISLDSSNASKRPCLPSVNNTGSINTNNVRKPKSQCKN  360
UWOPS05_227_2   301   SFNVVLPTNSNVTSSASSSTVSSISLDSSNASKRPCLPSVNNTGSINTNNVRKSKSQCKN  360
UWOPS05_217_3   301   SFNVVLPTNSNVTSSASSSTVSSISLDSSNASKRPCLPSVNNTGSINTNNVRKSKSQCKN  360
UWOPS03_461_4   301   SFNVVLPTNSNVTSSASSSTVSSISLDSSNASKRPCLPSVNNTGSINTNNVRKSKSQCKN  360
YPS606          301   SFNVVLPTNSNVTSSASSSTVSSISLDSSNASKRPCLPSVNNTGSINTNNVRKPKSQCKN  360
YPS128          301   SFNVVLPTNSNVTSSASSSTVSSISLDSSNASKRPCLPSVNNTGSINTNNVRKPKSQCKN  360
DBVPG6765       301   SFNVVLPTNSNVTSSASSSTVSSISLDSSNASKRPCLPSVNNTGSINTNNVRKPKSQCKN  360
YJM975          301   SFNVVLPTNSNVTSSASSSTVSSISLDSSNASKRPCLPSVNNTGSINTNNVRKPKSQCKN  360
BC187           301   SFNVVLPTNSNVTSSASSSTVSSISLDSSNASKRPCLPSVNNTGSINTNNVRKPKSQCKN  360
YJM981          301   SFNVVLPTNSNVTSSASSSTVSSISLDSSNASKRPCLPSVNNTGSINTNNVRKPKSQCKN  360
DBVPG1106       301   SFNVVLPTNSNVTSSASSSTVSSISLDSSNASKRPCLPSVNNTGSINTNNVRKPKSQCKN  360
DBVPG1373       301   SFNVVLPTNSNVTSSASSSTVSSISLDSSNASKRPCLPSVNNTGSINTNNVRKPKSQCKN  360
Y55             301   SFNVVLPTNSNVTSSASSSTVSSISLDSSNASKRPCLPSVNNTGSINTNNVRKPKSQCKN  360
YJM978          301   SFNVVLPTNSNVTSSASSSTVSSISLDSSNASKRPCLPSVNNTGSINTNNVRKPKSQCKN  360
L_1374          301   SFNVVLPTNSNVTSSASSSTVSSISLDSSNASKRPCLPSVNNTGSINTNNVRKPKSQCKN  360
L_1528          301   SFNVVLPTNSNVTSSASSSTVSSISLDSSNASKRPCLPSVNNTGSINTNNVRKPKSQCKN  360
DBVPG1788       301   SFNVVLPTNSNVTSSASSSTVSSISLDSSNASKRPCLPSVNNTGSINTNNVRKPKSQCKN  360
RM11_1A         301   SFNVVLPTNSNVTSSASSSTVSSISLDSSNASKRPCLPSVNNTGSINTNNVRKPKSQCKN  360
S. paradoxus    299   SFNVVLPTNSNVNSSASSSTVSSISLDSSNASKRPCLPPVNSTGSINTNNVRKPKSQCKN  358
```

Figure S1 Coding variants private to Malaysian yeast strains in iron-metabolism genes. Each panel shows the coding variants private to Malaysian yeast in one iron-metabolism protein, with the variants denoted as stars in a cartoon of the protein's domain organization at top, and a protein-coding alignment below for the region containing the Malaysian variant, using sequence and strain identifiers from (Liti *et al.* 2009). (A) Yap5p; (B) Ccc1p; (C) Aft1p. Non-synonymous coding variants private to the Malaysian population are indicated in red. bZIP_1, basic leucine zipper domain, PFAM clan 1; VIT1, vacuolar iron transport domain; AFT, activator of iron transcription domain; NLS, nuclear localization signal.

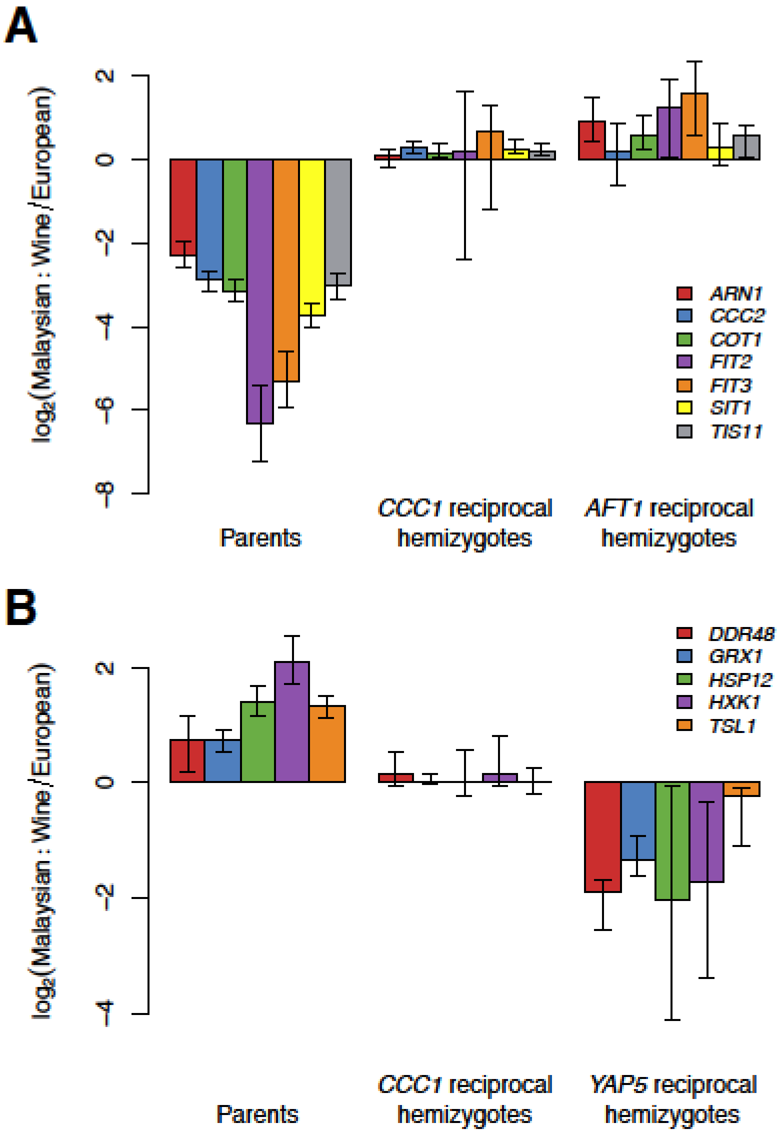

Figure S2 Regulatory impact of variation in *AFT1, CCC1,* and *YAP5* between Malaysian and wine/European yeast. Data are as in Figure 4 of the main text except that distributions of expression measurements across experimental replicates are shown. Each panel reports expression of iron-starvation (Gasch *et al.* 2004) and iron-resistance (Lin *et al.* 2011; Pimentel *et al.* 2012) genes as ratios of the levels measured in two strains derived from a Malaysian (UWOPS03.461.4) and a wine/European strain (BC187). At left in each row, each bar reports the median across replicates (n = 2) of the ratio of expression of the indicated gene between a Malaysian homozygote and a wine/European homozygote. In each remaining panel, each bar reports the median across replicates (*CCC1*, n = 8; *AFT1*, n = 4; *YAP5*, n = 4) of the ratio of expression of the indicated gene between a Malaysian-wine/European hemizygote bearing the Malaysian allele of the indicated variant locus, and the hemizygote bearing the wine/European allele. Error bars report 95% confidence intervals. (A) Iron-starvation genes in synthetic complete medium. (B) Iron-resistance genes in synthetic complete medium supplemented with 5 mM $FeSO_4$. Raw data are reported in Supplementary Data Sets 7 and 8.

Files S1-S8
Available for download at http://www.g3journal.org/lookup/suppl/doi:10.1534/g3.113.008011/-/DC1

File S1 Single-nucleotide polymorphisms in Malaysian and wine/European coding sequences inferred from RNA-seq. Each file reports single-nucleotide polymorphisms (SNPs) in open reading frames of one yeast strain, called with respect to the genome of the reference laboratory strain S288C (www.yeastgenome.org). In a given file, each row reports genotype information at one SNP position: the first column gives the chromosome identifier, the second column gives the position on the indicated chromosome, the third and fourth columns give the base present in the reference genome and the strain of interest, respectively, and the fifth column gives the Phred score of the genotype call.

File S2 Expression profiles of Malaysian and wine/European yeast, measured by RNA-seq. Each row reports a comparison of the expression of one gene between homozygote Malaysian (UWOPS03.461.4, UWOPS05.217.3, and UWOPS05.227.2) and wine/European (BC187 and RM11-1) yeast strains grown in standard culture conditions. For a given row, the second through sixth columns report raw counts of RNA-seq reads mapped to the indicated gene in libraries from the indicated strain. The remaining columns report the results of normalization and statistical testing from the DEseq software suite (Anders, Huber 2010): mean, average normalized expression of the indicated gene over all strains; WE_mean, average normalized expression across wine/European strains; M_mean, average normalized expression across Malaysian strains; foldChange and log2FoldChange, ratio and $\log_2$ ratio of the normalized mean expression in Malaysian strains relative to wine/European strains; pval, *p*-value assessing significance, in a negative binomial-based test, of the differential expression between populations; padj, corrected *p*-value by the Benjamini-Hochberg method.

File S3 Expression profiles of hybrid strains formed by a mating between Malaysian and wine/European yeast, measured by RNA-seq. Each row reports comparisons of the expression of the Malaysian allele of a gene to that of the wine/European allele of a gene, in hybrids formed by matings of Malaysian and wine/European strains. For a given gene, the second through sixth columns report raw counts of RNA-seq reads mapped to the indicated allele (M, Malaysian; WE, wine/European) in one hybrid strain, with identifiers as in Supplementary Table 2: YHL058, the Malaysian UWOPS03.461.4 mated to the wine/European RM11; YHL063, the Malaysian UWOPS03.461.4 mated to the wine/European BC187; YHL065, UWOPS05.217.3 mated to BC187. The remaining columns report the results of normalization and statistical testing from the DEseq software suite (Anders, Huber
2010): mean, average normalized expression of the indicated gene over all strains and alleles; WE_mean, average normalized expression across the wine/European alleles of all strains; M_mean, average normalized expression of the Malaysian alleles across all strains; foldChange and log2FoldChange, ratio and $\log_2$ ratio of the normalized mean expression of the Malaysian allele across all strains relative to that of the wine/European allele; pval, *p*-value assessing significance, in a negative binomial-based test, of differential allele-specific expression considering measures across the strains as replicates; padj, corrected *p*-value by the Benjamini-Hochberg method.

File S4 Directional *cis*-regulatory variation between Malaysian and wine/European yeast in co-regulated gene groups. Each row reports the results of a test for directional coherence of *cis*-regulatory variation between Malaysian and wine/European yeast in one group of functionally related genes, measured using reads uniquely mapped to each parent's allele of a given gene in turn, in hybrids formed by mating Malaysian and wine/European strains. Group, identifier of regulon from (Gasch et al. 2004) or Gene Ontology term. Upregulated, population with elevated expression; gene groups with an average expression difference of 0 between the populations are denoted with NA. Adjusted *p*-value, significance of a two-sided resampling test relative to the genomic null for an extreme value of the sum, across genes of the indicated regulon, of the $\log_2$ ratio of expression of the Malaysian allele of a given gene to expression of the wine/European allele, assessed using all isolates from each population and corrected for multiple testing with the Benjamini-Hochberg method. An additional test, not shown, for directional *cis*-regulatory variation across genes in a *ccc1* laboratory strain compared to wild-type during high-iron treatment (Lin et al. 2011) yielded a nominal *p*-value of 0.12.

File S5 Expression of iron-starvation genes in Malaysian and wine/European parent strains and reciprocal hemizygotes in standard conditions, measured by quantitative PCR. Each column reports comparisons between the effects of Malaysian and wine/European genotypes on expression of an iron-starvation gene during growth in standard medium. The first six rows report expression in a Malaysian (UWOPS03.461.4) and a wine/European strain (BC187). The remaining rows report expression in reciprocal hemizygote pairs in the UWOPS03.461.4 x BC187 background. Each identifier of the form *xxx/XXX*-Malaysian denotes a reciprocal hemizygote bearing only the Malaysian allele of the gene of interest, and *xxx/XXX*-wineEuropean denotes a hemizygote bearing only the wine/European allele. In row headings, each numerical value represents one biological replicate, and each row reports one technical replicate.

File S6 Expression of iron-resistance genes in Malaysian and wine/European parent strains and reciprocal hemizygotes in high-iron conditions, measured by quantitative PCR. Data are as in File S5 except that strains were cultured in standard medium supplemented with 5 mM $FeSO_4$, and iron-resistance gene expression was measured.

File S7 Growth attributes, in standard conditions and in high iron, of Malaysian and wine/European yeast, their hybrids, and reciprocal hemizygotes for *AFT1*, *YAP5*, and *CCC1*. Each row reports growth measurements, fitted to growth curves using the method of (Warringer et al. 2011), from one yeast culture in complete synthetic medium mock-treated (CSM) or treated with 5 mM $FeSO_4$. Strain names are as in Table S2. Hybrid, wild-type diploid formed by a mating between a Malaysian and a wine/European strain. Each identifier of the form *xxx/XXX*-Malaysian denotes a reciprocal hemizygote bearing only the Malaysian allele of the gene of interest, and *xxx/XXX*-wineEuropean denotes a hemizygote bearing only the wine/European allele. Doubling time, the inverse of the slope of a line fitted to the relationship between time (in hours) and cell density during log-phase growth; lag time, the *x*-intercept of a line fitted to the relationship between time (in hours) and cell density between inoculation and the onset of log-phase growth; efficiency, the ratio between the cell density at the end of a culture and the density at the start.

File S8 Growth attributes, in standard conditions and in high iron, of a panel of environmental yeast isolates. Data are as in File S7 except that wild-type isolates from each of the yeast populations defined in (Liti et al. 2009) were analyzed.

Table S1 RNA-seq statistics.

LIBRARY	TOTAL READS	UNIQUELY MAPPING READS
UWOPS03.461.4	70,275,289	37,992,498
UWOPS05.217.3	76,341,920	34,547,610
UWOPS05.227.2	63,801,895	28,127,756
BC187	62,813,354	35,442,506
RM11-1	76,501,254	35,055,758
UWOPS03.461.4 x BC187	137,307,729	28,763,569
UWOPS03.461.4 x RM11-1	164,905,032	31,783,714
UWOPS05.217.3 x BC187	47,399,026	6,133,068

Table S2 Strains used in this work.

STRAIN NAME	PARENTS	GENOTYPE	SOURCE
UWOPS03.461.4		*MATa/α HO*	NCYC
UWOPS05.217.3		*MATa/α HO*	NCYC
UWOPS05.227.2		*MATa/α HO*	NCYC
BC187		*MATa/α HO*	NCYC
UWOPS03.461.4 a/α		*MATa/α ura3Δ::kanMX hoΔ::hphMX*	NCYC
UWOPS05.217.3 a/α		*MATa/α ura3Δ::kanMX hoΔ::hphMX*	NCYC
UWOPS05.227.2 a/α		*MATa/α ura3Δ::kanMX hoΔ::hphMX*	NCYC
BC187 a/α		*MATa/α ura3Δ::kanMX hoΔ::hphMX*	NCYC
RM11-1	RM11-1a x RM11-1b	*MATa/α ura3 hoΔ::kanMX lys2/LYS2 leu2/LEU2*	Brem et al., 2002
YPS128		*MATa ura3Δ::kanMX hoΔ::hphMX*	NCYC
YPS606		*MATa ura3Δ::kanMX hoΔ::hphMX*	NCYC
Y12		*MATa ura3Δ::kanMX hoΔ::hphMX*	NCYC
NCYC110		*MATa ura3Δ::kanMX hoΔ::hphMX*	NCYC
S288c	BY4716 x BY4741	*MATa/α lys2/LYS2 leu2/LEU2 his3/HIS3 met15/MET15 ura3/URA3*	this study
UWOPS03.461.4 a		*MATa ura3Δ::kanMX hoΔ::hphMX*	NCYC
UWOPS03.461.4 α		*MATα ura3Δ::kanMX hoΔ::hphMX*	NCYC
UWOPS05.217.3 a		*MATa ura3Δ::kanMX hoΔ::hphMX*	NCYC
UWOPS05.217.3 α		*MATα ura3Δ::kanMX hoΔ::hphMX*	NCYC
BC187 a		*MATa ura3Δ::kanMX hoΔ::hphMX*	NCYC
BC187 α		*MATα ura3Δ::kanMX hoΔ::hphMX*	NCYC
RM11-1a		*MATa leu2 ura3 hoΔ::kanMX*	Brem et al., 2002
RM11-1b		*MATα lys2 ura3 hoΔ::kanMX*	Brem et al., 2002
YHL058	UWOPS03.461.4 x RM11-1b	*MATa/α HO/hoΔ::kanMX*	this study
YHL063	UWOPS03.461.4 x BC187	*MATa/α HO*	this study
YHL065	UWOPS05.217.3 x BC187	*MATa/α HO*	this study

YHL243	UWOPS03.461.4 α x BC187 a	*MATa/α ura3Δ::kanMX hoΔ::hphMX*	this study
YHL247	UWOPS03.461.4 a x BC187 α	*MATa/α ura3Δ::kanMX hoΔ::hphMX*	this study
YHL387	YHL243	*MATa/α ura3Δ::kanMX hoΔ::hphMX aft1Δ::URA3/AFT1-Malaysian*	this study
YHL388	YHL243	*MATa/α ura3Δ::kanMX hoΔ::hphMX aft1Δ::URA3/AFT1-Wine/European*	this study
YHL389	YHL243	*MATa/α ura3Δ::kanMX hoΔ::hphMX aft1Δ::URA3/AFT1-Malaysian*	this study
YHL409	YHL243	*MATa/α ura3Δ::kanMX hoΔ::hphMX yap5Δ::URA3/YAP5-Malaysian*	this study
YHL410	YHL243	*MATa/α ura3Δ::kanMX hoΔ::hphMX yap5Δ::URA3/YAP5-Malaysian*	this study
YHL411	YHL243	*MATa/α ura3Δ::kanMX hoΔ::hphMX yap5Δ::URA3/YAP5-WineEuropean*	this study
YHL413	YHL247	*MATa/α ura3Δ::kanMX hoΔ::hphMX yap5Δ::URA3/YAP5-Malaysian*	this study
YHL414	YHL247	*MATa/α ura3Δ::kanMX hoΔ::hphMX yap5Δ::URA3/YAP5-WineEuropean*	this study
YHL415	YHL247	*MATa/α ura3Δ::kanMX hoΔ::hphMX yap5Δ::URA3/YAP5-Malaysian*	this study
YHL449	YHL243	*MATa/α ura3Δ::kanMX hoΔ::hphMX ccc1Δ::URA3/CCC1-Wine/European*	this study
YHL450	YHL243	*MATa/α ura3Δ::kanMX hoΔ::hphMX ccc1Δ::URA3/CCC1-Malaysian*	this study
YHL451	YHL243	*MATa/α ura3Δ::kanMX hoΔ::hphMX ccc1Δ::URA3/CCC1-Malaysian*	this study
YHL459	YHL243	*MATa/α ura3Δ::kanMX hoΔ::hphMX ccc1Δ::URA3/CCC1-Wine/European*	this study
YHL452	YHL247	*MATa/α ura3Δ::kanMX hoΔ::hphMX ccc1Δ::URA3/CCC1-Wine/European*	this study
YHL455	YHL243	*MATa/α ura3Δ::kanMX hoΔ::hphMX aft1Δ::URA3/AFT1-Wine/European*	this study
