# Supplementary material for: Divergence of Iron Metabolism in Wild Malaysian Yeast
Source: G3 (Bethesda). 2013 Oct 18;3(12):2187–94. doi: 10.1534/g3.113.008011 (PMC3852381; doi:10.1534/g3.113.008011)
Supplement: Supporting Information [file supp_g3.113.008011_FigureS1.pdf]

A

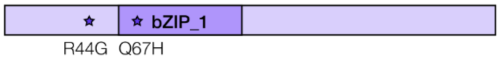


```
YPS606            21   KIHVSKNWKLPPRLPHRAAQRRKRVHRLHEDYETEENDEELQKKKRQNRDAQRAYRERKN   80
Y12               21   KIHVSKNWKLPPRLPHRAAQRRKRVHRLHEDYETEENDEELQKKKRQNRDAQRAYRERKN   80
YPS128            21   KIHVSKNWKLPPRLPHRAAQRRKRVHRLHEDYETEENDEELQKKKRQNRDAQRAYRERKN   80
Y9                21   KIHVSKNWKLPPRLPHRAAQRRKRVHRLHEDYETEENDEELQKKKRQNRDAQRAYRERKN   80
DBVPG6765         21   KIHVSKNWKLPPRLPHRAAQRRKRVHRLHEDYETEENDEELQKKKRQNRDAQRAYRERKN   80
BC187             21   KIHVSKNWKLPPRLPHRAAQRRKRVHRLHEDYETEENDEELQKKKRQNRDAQRAYRERKN   80
YJM981            21   KIHVSKNWKLPPRLPHRAAQRRKRVHRLHEDYETEENDEELQKKKRQNRDAQRAYRERKN   80
DBVPG1106         21   KIHVSKNWKLPPRLPHRAAQRRKRVHRLHEDYETEENDEELQKKKRQNRDAQRAYRERKN   80
DBVPG1373         21   KIHVSKNWKLPPRLPHRAAQRRKRVHRLHEDYETEENDEELQKKKRQNRDAQRAYRERKN   80
YJM978            21   KIHVSKNWKLPPRLPHRAAQRRKRVHRLHEDYETEENDEELQKKKRQNRDAQRAYRERKN   80
S288c             21   KIHVSKNWKLPPRLPHRAAQRRKRVHRLHEDYETEENDEELQKKKRQNRDAQRAYRERKN   80
YJM975            21   KIHVSKNWKLPPRLPHRAAQRRKRVHRLHEDYETEENDEELQKKKRQNRDAQRAYRERKN   80
L_1528            21   KIHVSKNWKLPPRLPHRAAQRRKRVHRLHEDYETEENDEELQKKKRQNRDAQRAYRERKN   80
RM11_1A           21   KIHVSKNWKLPPRLPHRAAQRRKRVHRLHEDYETEENDEELQKKKRQNRDAQRAYRERKN   80
DBVPG1788         21   KIHVSKNWKLPPRLPRRAAQRRKRVHRLHEDYETEENDEELQKKKRQNRDAQRAYRERKN   80
L_1374            21   KIHVSKNWKLPPRLPRRAAQRRKRVHRLHEDYETEENDEELQKKKRQNRDAQRAYRERKN   80
UWOPS05_227_2     21   KIHVSKNWKLPPRLPHRAAQRRKGVHRLHEDYETEENDEELQKKKRHNRDAQRAYRERKN   80
UWOPS05_217_3     21   KIHVSKNWKLPPRLPHRAAQRRKGVHRLHEDYETEENDEELQKKKRHNRDAQRAYRERKN   80
UWOPS03_461_4     21   KIHVSKNWKLPPRLPHRAAQRRKGVHRLHEDYETEENDEELQKKKRHNRDAQRAYRERKN   80
DBVPG6044         21   KIHVSKNWKLPPRLPHRAAQRRKRVHRLHEDYETEENDEELQKKKRQNRDAQRAYRERQN   80
NCYC110           21   KIHVSKNWKLPPRLPHRAAQRRKRVHRLHEDYETEENDEELQKKKRQNRDAQRAYRERQN   80
S. paradoxus      21   KIHVSKNWKLPPRLPHRATQRRKRAHRLHEEYETEGNDEALQKKKRQNRDAQRAYRERKN   80
```

B

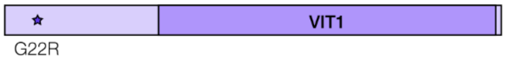


```
UWOPS05_227_2      1   MSIVALKNAVVTLIQKAKGSGRTSELGGSESTPLLRGSNSNSSRHDNLSSSSSDIIYGRN   60
UWOPS05_217_3      1   MSIVALKNAVVTLIQKAKGSGRTSELGGSESTPLLRGSNSNSSRHDNLSSSSSDIIYGRN   60
UWOPS03_461_4      1   MSIVALKNAVVTLIQKAKGSGRTSELGGSESTPLLRGSNSNSSRHDNLSSSSSDIIYGRN   60
DBVPG6765          1   MSIVALKNAVVTLIQKAKGSGGTSELGGSESTPLLRGSNSNSSRHDNLSSSSSDIIYGRN   60
DBVPG1788          1   MSIVALKNAVVTLIQKAKGSGGTSELGGSESTPLLRGSNSNSSRHDNLSSSSSDIIYGRN   60
RM11_1A            1   MSIVALKNAVVTLIQKAKGSGGTSELGGSESTPLLRGSNSNSSRHDNLSSSSSDIIYGRN   60
L_1528             1   MSIVALKNAVVTLIQKAKGSGGTSELGGSESTPLLRGSNSNSSRHDNLSSSSSDIIYGRN   60
YJM789             1   MSIVALKNAVVTLIQKAKGSGGTSELGGSESTPLLRGSNSNSSRHDNLSSSSSDIIYGRN   60
YJM975             1   MSIVALKNAVVTLIQKAKGSGGTSELGGSESTPLLRGSNSNSSRHDNLSSSSSDIIYGRN   60
L_1374             1   MSIVALKNAVVTLIQKAKGSGGTSELGGSESTPLLRGSNSNSSRHDNLSSSSSDIIYGRN   60
S288c              1   MSIVALKNAVVTLIQKAKGSGGTSELGGSESTPLLRGSNSNSSRHDNLSSSSSDIIYGRN   60
YPS606             1   MSIVALKNAVVTLIQKAKGSGGTSELGGSESTPLLRGSNSNSSRHDNLSSSSSDIIYGRN   60
Y12                1   MSIVALKNAVVTLIQKAKGSGGTSELGGSESTPLLRGSNSNSSRHDNLSSSSSDIIYGRN   60
YPS128             1   MSIVALKNAVVTLIQKAKGSGGTSELGGSESTPLLRGSNSNSSRHDNLSSSSSDIIYGRN   60
YJM978             1   MSIVALKNAVVTLIQKAKGSGGTSELGGSESTPLLRGSNSNSSRHDNLSSSSSDIIYGRN   60
Y9                 1   MSIVALKNAVVTLIQKAKGSGGTSELGGSESTPLLRGSNSNSSRHDNLSSSSSDIIYGRN   60
NCYC110            1   MSIVALKNAVVTLIQKAKGSGGTSELGGSESTPLLRGSNSNSSRHDNLSSSSSDIIYGRN   60
DBVPG1373          1   MSIVALKNAVVTLIQKAKGSGGTSELGGSESTPLLRGSNSNSSRHDNLSSSSSDIIYGRN   60
YJM981             1   MSIVALKNAVVTLIQKAKGSGGTSELGGSESTPLLRGSNSNSSRHDNLSSSSSDIIYGRN   60
BC187              1   MSIVALKNAVVTLIQKAKGSGGTSELGGSESTPLLRGSNSNSSRHDNLSSSSSDIIYGRN   60
DBVPG1106          1   MSIVALKNAVVTLIQKAKGSGGTSELGGSESTPLLRGSNSNSSRHDNLSSSSSDIIYGRN   60
DBVPG6044          1   MSIVALKNAVVTLIQKAKGSGGTSELGGSESTPLLRGSNSNSSRHDNLSSSSSDIIYGRN   60
S. paradoxus       1   MSIVALKNAVVTLIQKAKGSGGTSDLGGSESTPLLRGSNSNSSRHDNLSSSNSDIIYGRN   60
```

C

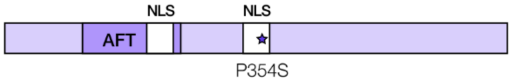


```
K11              301   SFNVVLPTNSNVTSSASSSTVSSISLDSSNASKRPCLPSVNNTGSINTNNVRKPKSQCKN  360
Y12              301   SFNVVLPTNSNVTSSASSSTVSSISLDSSNASKRPCLPSVNNTGSINTNNVRKPKSQCKN  360
Y9               301   SFNVVLPTNSNVTSSASSSTVSSISLDSSNASKRPCLPSVNNTGSINTNNVRKPKSQCKN  360
NCYC110          301   SFNVVLPTNSNVTSSASSSTVSSISLDSSNASKRPCLPSVNNTGSINTNNVRKPKSQCKN  360
DBVPG6044        301   SFNVVLPTNSNVTSSASSSTVSSISLDSSNASKRPCLPSVNNTGSINTNNVRKPKSQCKN  360
UWOPS05_227_2    301   SFNVVLPTNSNVTSSASSSTVSSISLDSSNASKRPCLPSVNNTGSINTNNVRKSKSQCKN  360
UWOPS05_217_3    301   SFNVVLPTNSNVTSSASSSTVSSISLDSSNASKRPCLPSVNNTGSINTNNVRKSKSQCKN  360
UWOPS03_461_4    301   SFNVVLPTNSNVTSSASSSTVSSISLDSSNASKRPCLPSVNNTGSINTNNVRKSKSQCKN  360
YPS606           301   SFNVVLPTNSNVTSSASSSTVSSISLDSSNASKRPCLPSVNNTGSINTNNVRKPKSQCKN  360
YPS128           301   SFNVVLPTNSNVTSSASSSTVSSISLDSSNASKRPCLPSVNNTGSINTNNVRKPKSQCKN  360
DBVPG6765        301   SFNVVLPTNSNVTSSASSSTVSSISLDSSNASKRPCLPSVNNTGSINTNNVRKPKSQCKN  360
YJM975           301   SFNVVLPTNSNVTSSASSSTVSSISLDSSNASKRPCLPSVNNTGSINTNNVRKPKSQCKN  360
BC187            301   SFNVVLPTNSNVTSSASSSTVSSISLDSSNASKRPCLPSVNNTGSINTNNVRKPKSQCKN  360
YJM981           301   SFNVVLPTNSNVTSSASSSTVSSISLDSSNASKRPCLPSVNNTGSINTNNVRKPKSQCKN  360
DBVPG1106        301   SFNVVLPTNSNVTSSASSSTVSSISLDSSNASKRPCLPSVNNTGSINTNNVRKPKSQCKN  360
DBVPG1373        301   SFNVVLPTNSNVTSSASSSTVSSISLDSSNASKRPCLPSVNNTGSINTNNVRKPKSQCKN  360
Y55              301   SFNVVLPTNSNVTSSASSSTVSSISLDSSNASKRPCLPSVNNTGSINTNNVRKPKSQCKN  360
YJM978           301   SFNVVLPTNSNVTSSASSSTVSSISLDSSNASKRPCLPSVNNTGSINTNNVRKPKSQCKN  360
L_1374           301   SFNVVLPTNSNVTSSASSSTVSSISLDSSNASKRPCLPSVNNTGSINTNNVRKPKSQCKN  360
L_1528           301   SFNVVLPTNSNVTSSASSSTVSSISLDSSNASKRPCLPSVNNTGSINTNNVRKPKSQCKN  360
DBVPG1788        301   SFNVVLPTNSNVTSSASSSTVSSISLDSSNASKRPCLPSVNNTGSINTNNVRKPKSQCKN  360
RM11_1A          301   SFNVVLPTNSNVTSSASSSTVSSISLDSSNASKRPCLPSVNNTGSINTNNVRKPKSQCKN  360
S. paradoxus     299   SFNVVLPTNSNVNSSASSSTVSSISLDSSNASKRPCLPPVNSTGSINTNNVRKPKSQCKN  358
```

Figure S1 Coding variants private to Malaysian yeast strains in iron-metabolism genes. Each panel shows the coding variants private to Malaysian yeast in one iron-metabolism protein, with the variants denoted as stars in a cartoon of the protein's domain organization at top, and a protein-coding alignment below for the region containing the Malaysian variant, using sequence and strain identifiers from (LITI *et al.* 2009). (A) Yap5p; (B) Ccc1p; (C) Aft1p. Non-synonymous coding variants private to the Malaysian population are indicated in red. bZIP_1, basic leucine zipper domain, PFAM clan 1; VIT1, vacuolar iron transport domain; AFT, activator of iron transcription domain; NLS, nuclear localization signal.
