# Supplementary material for: Divergence of Iron Metabolism in Wild Malaysian Yeast
Source: G3 (Bethesda). 2013 Oct 18;3(12):2187–94. doi: 10.1534/g3.113.008011 (PMC3852381; doi:10.1534/g3.113.008011)
Supplement: Supporting Information [file supp_g3.113.008011_FigureS2.pdf]

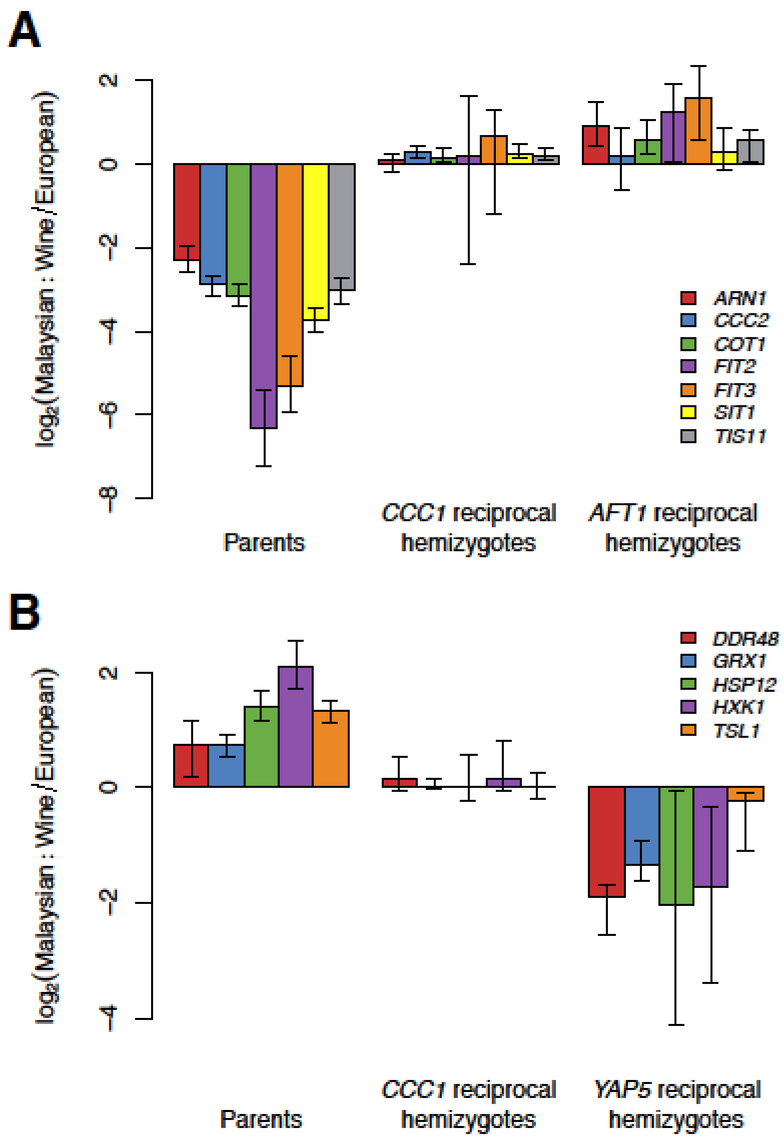

Figure S2 Regulatory impact of variation in *AFT1, CCC1,* and *YAP5* between Malaysian and wine/European yeast. Data are as in Figure 4 of the main text except that distributions of expression measurements across experimental replicates are shown. Each panel reports expression of iron-starvation (Gasch *et al.* 2004) and iron-resistance (Lin *et al.* 2011; Pimentel *et al.* 2012) genes as ratios of the levels measured in two strains derived from a Malaysian (UWOPS03.461.4) and a wine/European strain (BC187). At left in each row, each bar reports the median across replicates (n = 2) of the ratio of expression of the indicated gene between a Malaysian homozygote and a wine/European homozygote. In each remaining panel, each bar reports the median across replicates (*CCC1*, n = 8; *AFT1*, n = 4; *YAP5*, n = 4) of the ratio of expression of the indicated gene between a Malaysian-wine/European hemizygote bearing the Malaysian allele of the indicated variant locus, and the hemizygote bearing the wine/European allele. Error bars report 95% confidence intervals. (A) Iron-starvation genes in synthetic complete medium. (B) Iron-resistance genes in synthetic complete medium supplemented with 5 mM $FeSO_4$. Raw data are reported in Supplementary Data Sets 7 and 8.
