# Supplementary material for: Divergence of Iron Metabolism in Wild Malaysian Yeast
Source: G3 (Bethesda). 2013 Oct 18;3(12):2187–94. doi: 10.1534/g3.113.008011 (PMC3852381; doi:10.1534/g3.113.008011)
Supplement: Supporting Information [file supp_g3.113.008011_TableS1.pdf]

Table S1 RNA-seq statistics.

LIBRARY	TOTAL READS	UNIQUELY MAPPING READS
UWOPS03.461.4	70,275,289	37,992,498
UWOPS05.217.3	76,341,920	34,547,610
UWOPS05.227.2	63,801,895	28,127,756
BC187	62,813,354	35,442,506
RM11-1	76,501,254	35,055,758
UWOPS03.461.4 x BC187	137,307,729	28,763,569
UWOPS03.461.4 x RM11-1	164,905,032	31,783,714
UWOPS05.217.3 x BC187	47,399,026	6,133,068
