# Supplementary material for: Divergence of Iron Metabolism in Wild Malaysian Yeast
Source: G3 (Bethesda). 2013 Oct 18;3(12):2187–94. doi: 10.1534/g3.113.008011 (PMC3852381; doi:10.1534/g3.113.008011)
Supplement: Supporting Information [file supp_g3.113.008011_TableS2.pdf]

Table S2 Strains used in this work.

STRAIN NAME	PARENTS	GENOTYPE	SOURCE
UWOPS03.461.4		*MATa/α HO*	NCYC
UWOPS05.217.3		*MATa/α HO*	NCYC
UWOPS05.227.2		*MATa/α HO*	NCYC
BC187		*MATa/α HO*	NCYC
UWOPS03.461.4 a/α		*MATa/α ura3Δ::kanMX hoΔ::hphMX*	NCYC
UWOPS05.217.3 a/α		*MATa/α ura3Δ::kanMX hoΔ::hphMX*	NCYC
UWOPS05.227.2 a/α		*MATa/α ura3Δ::kanMX hoΔ::hphMX*	NCYC
BC187 a/α		*MATa/α ura3Δ::kanMX hoΔ::hphMX*	NCYC
RM11-1	RM11-1a x RM11-1b	*MATa/α ura3 hoΔ::kanMX lys2/LYS2 leu2/LEU2*	Brem et al., 2002
YPS128		*MATa ura3Δ::kanMX hoΔ::hphMX*	NCYC
YPS606		*MATa ura3Δ::kanMX hoΔ::hphMX*	NCYC
Y12		*MATa ura3Δ::kanMX hoΔ::hphMX*	NCYC
NCYC110		*MATa ura3Δ::kanMX hoΔ::hphMX*	NCYC
S288c	BY4716 x BY4741	*MATa/α lys2/LYS2 leu2/LEU2 his3/HIS3 met15/MET15 ura3/URA3*	this study
UWOPS03.461.4 a		*MATa ura3Δ::kanMX hoΔ::hphMX*	NCYC
UWOPS03.461.4 α		*MATα ura3Δ::kanMX hoΔ::hphMX*	NCYC
UWOPS05.217.3 a		*MATa ura3Δ::kanMX hoΔ::hphMX*	NCYC
UWOPS05.217.3 α		*MATα ura3Δ::kanMX hoΔ::hphMX*	NCYC
BC187 a		*MATa ura3Δ::kanMX hoΔ::hphMX*	NCYC
BC187 α		*MATα ura3Δ::kanMX hoΔ::hphMX*	NCYC
RM11-1a		*MATa leu2 ura3 hoΔ::kanMX*	Brem et al., 2002
RM11-1b		*MATα lys2 ura3 hoΔ::kanMX*	Brem et al., 2002
YHL058	UWOPS03.461.4 x RM11-1b	*MATa/α HO/hoΔ::kanMX*	this study
YHL063	UWOPS03.461.4 x BC187	*MATa/α HO*	this study
YHL065	UWOPS05.217.3 x BC187	*MATa/α HO*	this study

YHL243	UWOPS03.461.4 α x BC187 a	*MATa/α ura3Δ::kanMX hoΔ::hphMX*	this study
YHL247	UWOPS03.461.4 a x BC187 α	*MATa/α ura3Δ::kanMX hoΔ::hphMX*	this study
YHL387	YHL243	*MATa/α ura3Δ::kanMX hoΔ::hphMX aft1Δ::URA3/AFT1-Malaysian*	this study
YHL388	YHL243	*MATa/α ura3Δ::kanMX hoΔ::hphMX aft1Δ::URA3/AFT1-Wine/European*	this study
YHL389	YHL243	*MATa/α ura3Δ::kanMX hoΔ::hphMX aft1Δ::URA3/AFT1-Malaysian*	this study
YHL409	YHL243	*MATa/α ura3Δ::kanMX hoΔ::hphMX yap5Δ::URA3/YAP5-Malaysian*	this study
YHL410	YHL243	*MATa/α ura3Δ::kanMX hoΔ::hphMX yap5Δ::URA3/YAP5-Malaysian*	this study
YHL411	YHL243	*MATa/α ura3Δ::kanMX hoΔ::hphMX yap5Δ::URA3/YAP5-WineEuropean*	this study
YHL413	YHL247	*MATa/α ura3Δ::kanMX hoΔ::hphMX yap5Δ::URA3/YAP5-Malaysian*	this study
YHL414	YHL247	*MATa/α ura3Δ::kanMX hoΔ::hphMX yap5Δ::URA3/YAP5-WineEuropean*	this study
YHL415	YHL247	*MATa/α ura3Δ::kanMX hoΔ::hphMX yap5Δ::URA3/YAP5-Malaysian*	this study
YHL449	YHL243	*MATa/α ura3Δ::kanMX hoΔ::hphMX ccc1Δ::URA3/CCC1-Wine/European*	this study
YHL450	YHL243	*MATa/α ura3Δ::kanMX hoΔ::hphMX ccc1Δ::URA3/CCC1-Malaysian*	this study
YHL451	YHL243	*MATa/α ura3Δ::kanMX hoΔ::hphMX ccc1Δ::URA3/CCC1-Malaysian*	this study
YHL459	YHL243	*MATa/α ura3Δ::kanMX hoΔ::hphMX ccc1Δ::URA3/CCC1-Wine/European*	this study
YHL452	YHL247	*MATa/α ura3Δ::kanMX hoΔ::hphMX ccc1Δ::URA3/CCC1-Wine/European*	this study
YHL455	YHL243	*MATa/α ura3Δ::kanMX hoΔ::hphMX aft1Δ::URA3/AFT1-Wine/European*	this study
