# Supplementary material for: Divergence of Iron Metabolism in Wild Malaysian Yeast
Source: G3 (Bethesda). 2013 Oct 18;3(12):2187–94. doi: 10.1534/g3.113.008011 (PMC3852381; doi:10.1534/g3.113.008011)
Supplement: Supporting Information [file supp_g3.113.008011_FilesS1-S8.pdf]

Files S1-S8

Available for download at http://www.g3journal.org/lookup/suppl/doi:10.1534/g3.113.008011/-/DC1

File S1 Single-nucleotide polymorphisms in Malaysian and wine/European coding sequences inferred from RNA-seq. Each file reports single-nucleotide polymorphisms (SNPs) in open reading frames of one yeast strain, called with respect to the genome of the reference laboratory strain S288C (www.yeastgenome.org). In a given file, each row reports genotype information at one SNP position: the first column gives the chromosome identifier, the second column gives the position on the indicated chromosome, the third and fourth columns give the base present in the reference genome and the strain of interest, respectively, and the fifth column gives the Phred score of the genotype call.

File S2 Expression profiles of Malaysian and wine/European yeast, measured by RNA-seq. Each row reports a comparison of the expression of one gene between homozygote Malaysian (UWOPS03.461.4, UWOPS05.217.3, and UWOPS05.227.2) and wine/European (BC187 and RM11-1) yeast strains grown in standard culture conditions. For a given row, the second through sixth columns report raw counts of RNA-seq reads mapped to the indicated gene in libraries from the indicated strain. The remaining columns report the results of normalization and statistical testing from the DEseq software suite (Anders, Huber 2010): mean, average normalized expression of the indicated gene over all strains; WE_mean, average normalized expression across wine/European strains; M_mean, average normalized expression across Malaysian strains; foldChange and log2FoldChange, ratio and $\log_2$ ratio of the normalized mean expression in Malaysian strains relative to wine/European strains; pval, *p*-value assessing significance, in a negative binomial-based test, of the differential expression between populations; padj, corrected *p*-value by the Benjamini-Hochberg method.

File S3 Expression profiles of hybrid strains formed by a mating between Malaysian and wine/European yeast, measured by RNA-seq. Each row reports comparisons of the expression of the Malaysian allele of a gene to that of the wine/European allele of a gene, in hybrids formed by matings of Malaysian and wine/European strains. For a given gene, the second through sixth columns report raw counts of RNA-seq reads mapped to the indicated allele (M, Malaysian; WE, wine/European) in one hybrid strain, with identifiers as in Supplementary Table 2: YHL058, the Malaysian UWOPS03.461.4 mated to the wine/European RM11; YHL063, the Malaysian UWOPS03.461.4 mated to the wine/European BC187; YHL065, UWOPS05.217.3 mated to BC187. The remaining columns report the results of normalization and statistical testing from the DEseq software suite (Anders, Huber
2010): mean, average normalized expression of the indicated gene over all strains and alleles; WE_mean, average normalized expression across the wine/European alleles of all strains; M_mean, average normalized expression of the Malaysian alleles across all strains; foldChange and log2FoldChange, ratio and $\log_2$ ratio of the normalized mean expression of the Malaysian allele across all strains relative to that of the wine/European allele; pval, *p*-value assessing significance, in a negative binomial-based test, of differential allele-specific expression considering measures across the strains as replicates; padj, corrected *p*-value by the Benjamini-Hochberg method.

File S4 Directional *cis*-regulatory variation between Malaysian and wine/European yeast in co-regulated gene groups. Each row reports the results of a test for directional coherence of *cis*-regulatory variation between Malaysian and wine/European yeast in one group of functionally related genes, measured using reads uniquely mapped to each parent's allele of a given gene in turn, in hybrids formed by mating Malaysian and wine/European strains. Group, identifier of regulon from (Gasch et al. 2004) or Gene Ontology term. Upregulated, population with elevated expression; gene groups with an average expression difference of 0 between the populations are denoted with NA. Adjusted *p*-value, significance of a two-sided resampling test relative to the genomic null for an extreme value of the sum, across genes of the indicated regulon, of the $\log_2$ ratio of expression of the Malaysian allele of a given gene to expression of the wine/European allele, assessed using all isolates from each population and corrected for multiple testing with the Benjamini-Hochberg method. An additional test, not shown, for directional *cis*-regulatory variation across genes in a *ccc1* laboratory strain compared to wild-type during high-iron treatment (Lin et al. 2011) yielded a nominal *p*-value of 0.12.

File S5 Expression of iron-starvation genes in Malaysian and wine/European parent strains and reciprocal hemizygotes in standard conditions, measured by quantitative PCR. Each column reports comparisons between the effects of Malaysian and wine/European genotypes on expression of an iron-starvation gene during growth in standard medium. The first six rows report expression in a Malaysian (UWOPS03.461.4) and a wine/European strain (BC187). The remaining rows report expression in reciprocal hemizygote pairs in the UWOPS03.461.4 x BC187 background. Each identifier of the form *xxx/XXX*-Malaysian denotes a reciprocal hemizygote bearing only the Malaysian allele of the gene of interest, and *xxx/XXX*-wineEuropean denotes a hemizygote bearing only the wine/European allele. In row headings, each numerical value represents one biological replicate, and each row reports one technical replicate.

File S6 Expression of iron-resistance genes in Malaysian and wine/European parent strains and reciprocal hemizygotes in high-iron conditions, measured by quantitative PCR. Data are as in File S5 except that strains were cultured in standard medium supplemented with 5 mM $FeSO_4$, and iron-resistance gene expression was measured.

File S7 Growth attributes, in standard conditions and in high iron, of Malaysian and wine/European yeast, their hybrids, and reciprocal hemizygotes for *AFT1*, *YAP5*, and *CCC1*. Each row reports growth measurements, fitted to growth curves using the method of (Warringer et al. 2011), from one yeast culture in complete synthetic medium mock-treated (CSM) or treated with 5 mM $FeSO_4$. Strain names are as in Table S2. Hybrid, wild-type diploid formed by a mating between a Malaysian and a wine/European strain. Each identifier of the form *xxx/XXX*-Malaysian denotes a reciprocal hemizygote bearing only the Malaysian allele of the gene of interest, and *xxx/XXX*-wineEuropean denotes a hemizygote bearing only the wine/European allele. Doubling time, the inverse of the slope of a line fitted to the relationship between time (in hours) and cell density during log-phase growth; lag time, the *x*-intercept of a line fitted to the relationship between time (in hours) and cell density between inoculation and the onset of log-phase growth; efficiency, the ratio between the cell density at the end of a culture and the density at the start.

File S8 Growth attributes, in standard conditions and in high iron, of a panel of environmental yeast isolates. Data are as in File S7 except that wild-type isolates from each of the yeast populations defined in (Liti et al. 2009) were analyzed.
